# Horizontal transfer and evolution of transposable elements in vertebrates

**DOI:** 10.1038/s41467-020-15149-4

**Published:** 2020-03-13

**Authors:** Hua-Hao Zhang, Jean Peccoud, Min-Rui-Xuan Xu, Xiao-Gu Zhang, Clément Gilbert

**Affiliations:** 1 College of Pharmacy and Life Science, Jiujiang University, 332000 Jiujiang, China; 2 UMR CNRS 7267 Ecologie et Biologie des Interactions, Equipe Ecologie Evolution Symbiose, Université de Poitiers, 86073 Poitiers, France; 3 Laboratoire Evolution, Génomes, Comportement, Écologie, UMR 9191 CNRS, UMR 247 IRD, Université Paris-Saclay, 91198 Gif-sur-Yvette, France

**Keywords:** Bioinformatics, Evolutionary genetics, Molecular evolution, Genome

## Abstract

Horizontal transfer of transposable elements (HTT) is an important process shaping eukaryote genomes, yet very few studies have quantified this phenomenon on a large scale or have evaluated the selective constraints acting on transposable elements (TEs) during vertical and horizontal transmission. Here we screen 307 vertebrate genomes and infer a minimum of 975 independent HTT events between lineages that diverged more than 120 million years ago. HTT distribution greatly differs from null expectations, with 93.7% of these transfers involving ray-finned fishes and less than 3% involving mammals and birds. HTT incurs purifying selection (conserved protein evolution) on all TEs, confirming that producing functional transposition proteins is required for a TE to invade new genomes. In the absence of HTT, DNA transposons appear to evolve neutrally within genomes, unlike most retrotransposons, which evolve under purifying selection. This selection regime indicates that proteins of most retrotransposon families tend to process their own encoding RNA (*cis*-preference), which helps retrotransposons to persist within host lineages over long time periods.

## Abstract (web summary)

Horizontal transfer (HT) and evolution of transposable elements (TEs) has rarely been quantified on a large scale. Here, the authors screen 307 vertebrate genomes and infer 975 HT events (93% in ray-finned fishes); all TEs involved in HT evolve within genomes under purifying selection, as do most retrotransposons.

## Introduction

Transposable elements (TEs) are pieces of DNA able to move from one locus to another in genomes, often duplicating themselves in the process^1^. This property has allowed TEs to invade the genomes of virtually all organisms, of which they can make up most of the chromosomes. Their activity and repeated nature have profoundly shaped the genomic architectures and phenotypes of diverse lineages^2–5^. Like any genetic component, TE copies can be inherited vertically, from parents to descendants. In addition, TEs can be transmitted from one organism to another in the absence of reproduction, through horizontal transfer (HT)^6–8^. The precise mechanisms and conditions of HT of TEs (HTT), although uncharacterized, most certainly relate to the inherent mobility of TEs. In comparison, regular genes are much more rarely found to be horizontally transferred in eukaryotes^9–14^. HTT is typically inferred when the nucleotide divergence between TE copies from two host lineages is much lower than what would be expected to result from vertical inheritance since the last common ancestor of the two hosts. The first HTT ever inferred in eukaryotes was that of the *P* element, which is thought to have been transferred horizontally from *Drosophila willistoni* to some populations of *D. melanogaster*^15^. Since then, hundreds of HTT events have been inferred in eukaryotes, showing that both DNA transposons (Class-II TEs) and retrotransposons (Class-I TEs) can be horizontally transferred, that HTT can involve a large variety of eukaryote lineages and that TEs can transfer both between closely and distantly related lineages^16^. In addition, a number of TEs inducing important phenotypic changes on their host are known to have been acquired through HT, setting HTT as a source of genetic variation fueling adaptive change^17^. Yet, HTT is still far from being well understood in eukaryotes. In particular, the frequency and impact of HTT that have occurred during the evolution of large clades, and the factors governing these transfers, are poorly known. This limited understanding is due in part from the reliance on manual curation of TEs and individual detection and validation of transfer events, which has restricted the scope of studies on HTT^18^. The lack of global quantification of HTT echoes the limited knowledge about the selective constraints acting on TEs during vertical versus horizontal transmission (but see ref. ^19–21^). In particular, one may explain the long-term persistence of certain retrotransposons in vertebrates^22^ as the outcome of selection of functional over non-functional TE copies during the reverse-transcription phase that occurs during transposition, independently of the effect of TEs on host fitness. Purifying selection should translate into conserved evolution of transposition proteins (lower rates of non-synonymous than synonymous mutations). However, this prediction has never been formally tested.

To fill gaps in our knowledge of TE HT and evolution, procedures have recently been developed for the automated detection and count of HTT events in hundreds of eukaryote genomes^23–26^. Two large-scale studies^24,25^ have applied these procedures to arthropods, hereby multiplying the number of identified HTT events. These large numbers have allowed us to establish a positive influence of phylogenetic and geographical proximity on HTT^24^ and to outline lepidoptera as particularly prone to transfer TEs of different classes among arthropods^25^. To our knowledge, however, no study has yet established the selection regimes acting on TEs at such a large scale. Here we adapt and improve on a procedure we previously developed^24^ to systematically detect and count HTT events among vertebrates, a large clade of more than 69,000 animal species that spans more than 500 million years of evolution^27^, and which is known to harbor a large diversity of TE families displaying contrasted evolutionary dynamics^28,29^. We leverage our HTT detection pipeline to unveil broad-scale patterns of selection acting on DNA transposons and retrotransposons during both transposition within host lineages and horizontal transfer. We show that at least 975 independent HTT events have punctuated vertebrate evolutionary history, mostly in ray-finned fishes and much more rarely in birds and mammals. We also show that HTT incurs purifying selection (conserved protein evolution) on all TE types, while DNA transposons evolve neutrally within genomes in the absence of HTT. By contrast, most retrotransposons diversify within genomes under purifying selection, which suggests that retrotransposon proteins tend to preferentially process and reverse transcribe their own encoding RNAs.

## Results and discussion

### Frequent horizontal transfers of transposable elements

We investigated HTT involving most TE types among 307 vertebrate species whose genome sequences are publicly available on GenBank (Supplementary Data 1), following principles developed in earlier studies^18,24^. To avoid detection biases that could have arisen from the use of available TE databases of varying quality across species, TEs were de novo characterized with the same procedure for all genomes. Consensus sequences (Supplementary Data 2) were built with the RepeatModeler pipeline^30^ and TE copies were annotated using RepeatMasker^31^. We excluded elements that might contain genes coding for proteins that were not listed in TE protein databases, as a result of erroneous TE superfamily assessment. More than 133 million TE copies ≥ 300 base pairs (bp) belonging to diverse superfamilies of retrotransposons and DNA transposons were extracted from all genomes (Supplementary Data 3).

We then performed similarity searches through reciprocal nucleotide blast^32^ comparing every TE copy of one species to another species’ copies. This involved 87,818 blast searches for all possible pairs of species that diverged before the last 40 million years. We considered that HTT between more recently diverged species could not be safely distinguished from vertical TE inheritance using an automated procedure. Among the ~108 million retained hits of sufficient sequence identity (≥75%) and alignment score (≥200), we selected those that may reflect HTT rather than vertical inheritance of TE copies from a common ancestor of the species. This selection was based on the premise that, TEs being horizontally transferred between species that have de facto already diverged, these TEs should show higher sequence identity than regular orthologous genes, which started diverging at the same time as their carrier species. We therefore selected hits involving copies whose synonymous sequence divergence at protein-coding regions (Ks) was lower than 99.5% of the Ks values of core orthologous genes from the vertebrate lineages involved in the hits. Despite these stringent filters, patterns of synonymous and non-synonymous divergence between TEs (Supplementary Fig. 1) suggested that some TE similarities between the most closely related species might have resulted from vertical inheritance of TEs rather than HTT. These patterns led us to exclude any hit involving species that diverged within the last 120 million years, which effectively removed all hits within eutherian mammals and birds. The remaining 876,528 hits were considered to result from HTT.

**Fig. 1:**
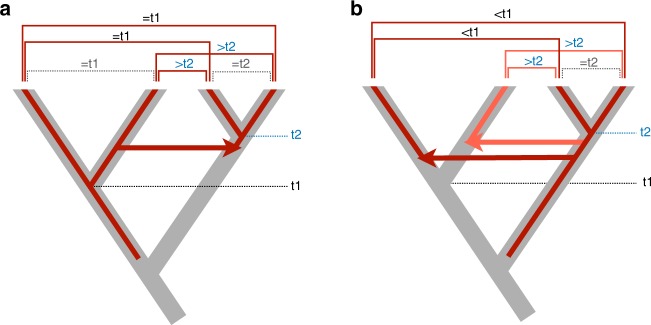
Inferring horizontal transfers between two animal clades from homologies (hits) between transposable element copies. Species trees are drawn with thick gray branches, red branches representing phylogenies of TEs with HTT events shown as horizontal arrows. Hits between TE copies are represented by horizontal square brackets above tree tips, those reflecting vertical inheritance of TEs (hits within clades) are represented as dashed brackets. t1 and t2 represent the divergence of species within clades. **a** A single HTT event should result in similarities between TEs of different clades (shown above hits) that are lower than the similarities of TEs (and of species, as estimated from synonymous divergence at orthologous genes) within at least one of the clades. **b** The divergence time (inferred from DNA sequence divergence) between TEs from the different clades is always lower than species divergence within the oldest clade (t1). These hits cannot result from a single HTT event, even though divergence of TE copies within the right-hand clade can be lower than that of copies between clades.

Importantly, the number of HTT events to explain these hits is considerably lower, since the transfer of only one functional TE between vertebrate lineages can yield thousands of similar copies that diverged from this initial TE through transposition within genomes and speciation within lineages. To account for these scenarios, we iteratively clustered blast hits involving species of the same pair of vertebrate clades into “hit groups” that represent separate HTT events^24^. In essence, for two hits between the same two vertebrate clades to be considered as resulting from a single transfer event, some of the TE copies they involve must have diverged within a recipient clade after the transfer. Hence, TE copies involved in the hits must show lower sequence identity within at least one of the clades than between clades. Furthermore, the degree of sequence identity of hits involving different species pairs must be compatible with the hypothesis of a transfer occurring before the divergence of recipient species (Fig. 1). Clustering of hits that passed these criteria resulted in 7940 hit groups. From these, we eliminated hit groups comprising less than five TE copies in either of the two clades involved in the transfer, to minimize the risk of taking DNA contamination in assembled genomes as HTT. Lastly, certain hit groups that might not result from HTT, based on the shape of the Ks distribution of their constituting hits, were removed (see Methods section).

**Fig. 2:**
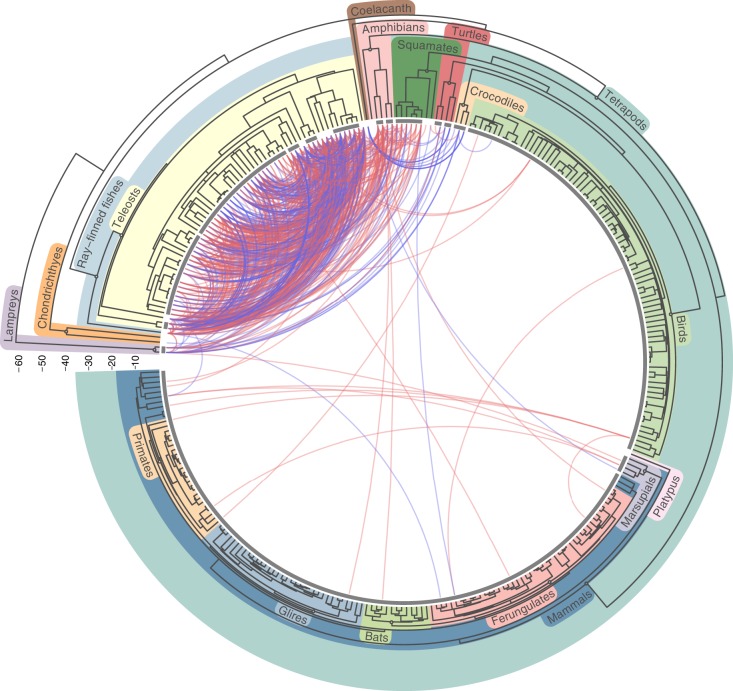
Horizontal transfer of transposable elements among vertebrates. The concave tree represents the time-based phylogeny of the 307 analyzed species and was retrieved from timetree.org (50). Each curve represents one of the 975 independent HTT events we inferred. It connects the two species involved in the hit of highest sequence identity in the transfer. Blue curves represent HTT of retrotransposons (Class-I TEs) and red curves HTT of DNA transposons (Class-II TEs). The horizontal scale on the left shows ages in 10 My. Gray lines above tips span clades that are less than 120 My old, within which HTT was not inferred.

These filters yielded a final number of 2632 hit groups constituting 775,801 TE–TE hits (Supplementary Data 4). While any two hit groups must represent at least two separate horizontal transfers, three or more hit groups may not represent as many events. Indeed, the presence of similar TEs in distantly related species need not reflect a direct transfer from one to the other, but independent incoming transfers of these TEs from other sources (a hit group is referred to as an “indirect transfer” in this case). To estimate the minimal number of independent transfer events required to explain the data, we investigated whether TEs composing each hit group could have been brought into their host species via other transfers already represented by other such groups. This analysis yielded 975 independent HTT events across the 307 genomes (Fig. 2), a number that is more than ten times that of previously identified HTTs involving vertebrates^16^. Yet, we view this number as quite conservative as our extremely stringent filters likely discarded many hits that did not actually result from vertical TE inheritance, and our clustering approach may have aggregated independent HTT events into one. Our automatic annotation procedure may also have missed TEs present in genomes, thus we stress that the numbers we report only apply to elements that were successfully classified into superfamilies.

Interestingly, while the number of genomes we surveyed is more than 1.5 times that of an earlier study on HTT in insects^24^, we counted ~2.3 times fewer HTT events. Explaining this difference by biological factors would be hazardous, because the much higher synonymous mutation rates of insect genomes (~2.57 × 10^−8^ mutation/site/year^24^) compared to vertebrates (~3.1 × 10^−9^ mutation/site/year, Supplementary Fig. 2) allows the detection of HTT between much more closely related species^33,34^. In vertebrates, the strong suspicion that some DNA transposon copies were not horizontally transferred despite being less divergent than almost all vertically inherited genes of their host species (Supplementary Fig. 1) led us to ignore any homologies between TEs of species that diverged within the last 120 million years (My). Clearly, filters designed to select homologies resulting from HTT might pick up the few vertically inherited TEs copies that happened to have diverged at the slowest pace. As a result, HTT among species that are not sufficiently divergent on a molecular level will remain difficult to reliably ascertain^18^. This risk precluded the detection of HTT within mammals and birds.

### Excess of horizontal transfer of TEs among ray-finned fishes

**Fig. 3:**
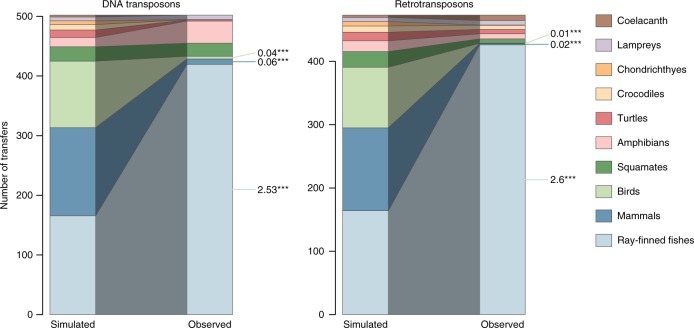
Contrasting simulated and observed distributions of HTT events involving different vertebrate clades, for the two classes of transposable elements. The left-hand barplot of each graph represents an average over distributions obtained by 1000 random permutations of species involved in HTT. Asterisks indicate clades for which observed numbers of HTT were smaller or higher than those yielded by all permutations (*p* < 1‰), and numbers next to them are ratios between these observed and averaged simulated numbers of HTT events. For other clades, observed numbers were between the 2.5 and 97.5% quantiles of those yielded by permutations (*p* > 5%).

Ray-finned fishes (Actinopterygii) contribute the vast majority (~94%) of transfer events (Fig. 2) despite representing just 64 of the 307 studied genomes. By comparison, only 24 detected HTT events involve mammals and birds, even though they represent 218 species among the 307 we analyzed. Such contrast called for testing a relative excess of HTT events involving certain vertebrate clades, while taking into account the technical limitations we had to cope with. In a fashion similar to a previous study^25^, we generated null distributions of HTT events across taxa through random species permutation, i.e., by replacing species by others among those involved in transfers of a given TE superfamily, a procedure that maintains the relative numbers of HTT per species. We discarded any permutation leading to transfers between species that would have diverged within the last 120 My, since such transfers were not considered in real data. We found that the actual number of HTT events involving ray-finned fishes exceeded numbers yielded by all permutations for both TE classes (Fig. 3) and for every TE superfamily that constituted at least 20 transfers (Supplementary Table 1). On the opposite, the actual numbers of transfers involving mammals and birds was between ~16 to 100 times less than averages obtained through random permutations. These relative deficits cannot result from our filters that prevented the detection of HTT within these clades, since these filters are accounted for in our permutation procedure. It should however be stressed that reported excesses and deficits are only relative to each other and inter-dependent (excesses imply deficits and vice versa). Currently, no method can predict an absolute frequency of HTT events involving any lineage, as processes controlling HTT rates are largely unknown. We further emphasize that the relative deficits and excesses of HTT events only apply to the transfers that we were able to detect, i.e., those involving at least two vertebrate lineages that diverged before the last 120 million years. In particular, mammals and birds may frequently exchange TEs among themselves or with other organisms. A recent report^23^ of dozens of horizontal transfers of RTE-BovB retrotransposons between mammals and other vertebrates contrasts with our findings. We attribute this contrast to the conservativeness of our estimate of the number of HTT events (Supplementary Discussion) rather than a particular bias against the detection of HTT in mammals, since our selection criteria were independent of the vertebrate groups we investigated.

The heterogenous contribution of vertebrate clades to HTT reflects the results of another recent survey^25^ showing that HTT in arthropods preferentially involves lepidopterans (butterflies and moths). To explain this trend, it has been suggested that baculoviruses may shuttle TEs between lepidopteran hosts^25,35^. Similarly, ray-finned fishes could be part of ecological networks comprising organisms or environments that are particularly prone to shuttle TEs, such as viruses and other parasites^36,37^, and/or they may present physiological or biochemical properties that facilitate emission and/or acquisition of the type of TEs contributing to the transfers we detected. To better understand the departure of the observed HTT distribution from null expectations, we tested whether aquatic vertebrates were more likely to exchange TEs than terrestrial ones. Because this ecological trait is deeply coupled to the species phylogeny (only tetrapods can be terrestrial), we randomly swapped the habitat of tetrapod species, from aquatic to terrestrial or vice-versa (considering amphibious species as aquatic), and compared these randomized data to real ones in respect to the number of transfers that species from these two habitats had with ray-finned fishes. The actual numbers of HTT events between aquatic tetrapods and fishes were within the range (i.e., between the 2.5 and 97.5% quantiles) of numbers obtained from permutations. Consequently, there is little evidence that aquatic lifestyle generally facilitates the HTT we detected, keeping in mind that the low number of aquatic tetrapod lineages limits the power of our analysis. A more powerful test of the hypothesis that aquatic environments favor HTT would require aquatic and non-aquatic species of multiple evolutionary origins.

### Contributions of TE superfamilies to horizontal transfer

**Fig. 4:**
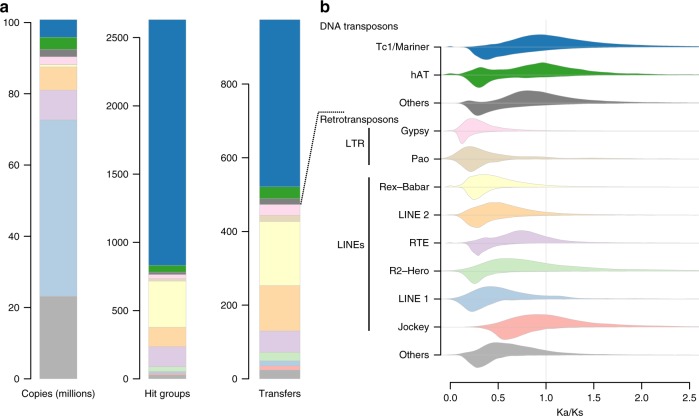
TEs superfamilies involved in horizontal transfer among the 307 studied vertebrate genomes. **a** Statistics on TEs superfamilies. Numbers of TE copies only consider those of at least 300 bp. “Hit groups” represent horizontal transfer events that may either be direct or indirect (see text). “Transfers” represent the minimal numbers of HTT events required to explain the data. Superfamilies are shown in the same colors as in (**b**). **b** Distributions of Ka/Ks ratios between TEs. For each TE superfamily, the upper curve shows the distribution of Ka/Ks ratios between TEs that diverged through transposition within genomes, and the lower curve represents Ka/Ks ratios between TEs that partly diverged through HTT (i.e., that belong to the different vertebrate clades involved in a transfer). Ka/Ks ratios are significantly lower than 1 for all distributions (*p* < 1%, one-sided Mann–Whitney *U*-tests, Supplementary Table 2), except for those of DNA transposons and Jockey retrotransposons that diverged through transposition only.

The most common type among horizontally transferred TEs appears to be DNA transposons of the Tc1/Mariner superfamily (Fig. 4a), in line with earlier results that consistently show prevalence of Tc1/Mariner TEs in HTT among diverse animals^21,24,25,34,38^. The high interspecific mobility of DNA transposons is consistent with the “blurry” promoters and low reliance on host factors enabling these TEs to easily transpose in a large panel of hosts^39–41^. The relative contribution of Tc1/Mariner TEs to HTT in vertebrates still seems lower than those reported by previous studies, which generally found DNA transposons to be much more frequently transferred than retrotransposons^7,24,42^. Noteworthily, DNA transposons, and Tc1/Mariner in particular, largely prevail among TEs composing hit groups, despite showing much lower proportion among independent HTT events (Fig. 4a). This discrepancy means that a hit group of Tc1/Mariner TEs was frequently inferred as an indirect transfer and was therefore not counted among independent HTT events. The frequency of this inference may be explained by the fact that Tc1/Mariner TEs from different hit groups tend to show similar DNA sequences, more so than most other TE superfamilies, and that (ii) many of the investigated species, especially fishes, share these TEs. Previous studies of HTT generally investigated far fewer genomes and/or TEs, and used different approaches to count transfer events. Hence, the effect we mention may have had less impact on the contribution of Tc1/Mariner TEs in HTT.

Besides Tc1/Mariner, the non-LTR retrotransposon Rex-Babar stands out as being the superfamily involved in the second highest number of transfers, with one of the highest ratios of transfers per copy. Most (~87.6%) HT events involving Rex-Babar TEs were detected among teleost fishes, in agreement with earlier studies showing that this superfamily was widespread in this clade and likely underwent horizontal transmission^43,44^. At the opposite side of the spectrum, the LINE 1 superfamily stands out as having generated the highest number of annotated copies overall, while seldom relying on HT to persist in its host lineages (Fig. 4a). This pattern remarkably echoes that emerging from earlier studies of these elements in vertebrates, suggesting that most lineages of LINE 1 currently found in vertebrate genomes result from long-term vertical transmission and co-evolution with their hosts^22,45^. In the same vein, the scarcity of detected HT involving endogenous retroviruses (ERVs) between lineages separated by more than 120 million years corroborates a previous large-scale study of these elements^46^, which showed that ERVs have undergone only few host switches between vertebrate classes.

### Selective constraints acting on transposable elements

Our global analysis of TEs among vertebrates offered the opportunity to investigate the extent to which their molecular evolution occurred under natural selection, on a large scale. We did so by establishing the ratios of non-synonymous (Ka) to synonymous (Ks) mutation rates of TE protein-coding regions. Although a TE protein is generally useless to an animal^47^, it may contribute to the transposition, hence to the replication and successful horizontal transfer, of its source TE copy. Broad signatures of selection on TE protein-coding sequences therefore represent variations in replication rates among TEs rather than the effects of these TEs on host fitness.

The degradation of TE protein-coding regions by random mutations (many of which cause frameshifts) and the sheer number of TE copies imposed great challenges to the analysis of Ka/Ks ratios. Our automatic procedure (detailed in the Methods section) was designed to overcome these challenges at the expense of measuring Ka and Ks mutation rates on pairwise, rather than on multiple, sequence alignments. We thus established Ka/Ks ratios from the 775,801 pairs of TE copies that underwent HT during their divergence (i.e., those involved in the retained hits) and ratios computed between related TE copies (constituting more than 4.6 million pairs of TEs) within the same genome and hit group, the divergence of which involves transposition only (Fig. 4b). These Ka/Ks ratios may be biased by certain factors that our pairwise approach cannot properly control for, namely mutational saturation and non-stationarity in DNA base composition^48,49^, and they do not account for changes in selection regimes during TE evolution, in particular the relaxing of selection after the potential TE deactivation. We however determined (Supplementary Discussion, Supplementary Figs. 3–6) that these potential effects cannot explain the variations of Ka/Ks ratios we report below.

Among TEs that diverged within genomes, Ka/Ks ratios are significantly < 1 (one-sided, one-sample Mann–Whitney tests, *p* < 1%, Supplementary Table 2) to the exceptions of DNA transposons and retrotransposons of the Jockey super family. To our knowledge, such patterns of purifying selection (conserved TE protein-sequence evolution) within an animal lineage have only been reported for the human LINE 1^20^ and endogenous retrovirus ERV-K elements^19^. In the human LINE 1, purifying selection can be explained by *cis*-preference^50^, i.e., the propensity of (de facto functional) retrotransposon proteins to process, hence to transpose and replicate, their own encoding mRNAs. Our findings suggest that *cis*-preference could be a feature of most vertebrate LINE super families. However, LTR retrotransposons are not known to display *cis*-preference^51^ and yet present the lowest Ka/Ks ratios (Fig. 4b and Supplementary Table 2). For these elements, the expression of a single LTR copy per cell at a time may constrain LTR proteins to replicate their own source mRNAs, even in the absence of *cis*-preference, as proposed for the human ERV-K^19^.

At the other end of the spectrum, Jockey elements and DNA transposons present Ka/Ks ratios that are not significantly lower than one (one-sided, one-sample Mann–Whitney tests, *p* > 5%, Supplementary Table 2), indicating that these TEs diversify within genomes under more relaxed selection than the others. Their replication would predominantly involve *trans*-complementation, whereby TE copies are transposed by proteins encoded by other copies^52,53^. For Jockey elements, it is difficult to exclude some degree of *cis*-preference, as our power to detect selection is limited (Supplementary Discussion). Regarding DNA transposons, their replication mechanism leaves no opportunity for *cis*-preference. A transposase has indeed no way to recognize its cognate copy, which is processed as a nuclear chromosomal segment and not as a cytoplasmic mRNA molecule.

While *trans*-complementation allows the replication of non-functional TEs within hosts, only those encoding functional proteins may successfully move between genetically divergent species if the recipient genomes lack the proteins that could transpose these TEs. Expectedly, TEs that diverge through HT show evidence for conserved protein evolution (Ka/Ks ratios < 1, Fig. 4b and Supplementary Table 2) in all super families, including Jockey and DNA transposons. These results generalize earlier findings obtained from the mellifera family of insect Mariner elements^21^ and show that DNA transposons evolve under different selecting pressures during horizontal transfer and within-genome transposition.

We do not exclude that a minority of TEs may evolve under selection regimes that we did not infer. For example, the coiled-coil domain of the primate LINE1 ORF1 is known to have evolved under positive selection (adaptive evolution) during part of its history^20^. As the inference of positive selection is more complex than that of purifying selection, new frameworks are needed to investigate positive selection at the scale of many host lineages and TE types.

## Conclusion

To conclude, our study reveals that the evolutionary history of TEs in vertebrates has been punctuated by a large number of HT events, most of which involved members of the Tc1/Mariner superfamily in ray-finned fishes. The emergence of lepidopterans^25^ and ray-finned fishes as hotspots of HTT sets host lineage as an important factor driving HTT among animals and raises the question as to why certain taxa are more prone to HTT than others. Our broad-scale analysis of the molecular evolution of vertebrate TEs also extends previous results showing that DNA transposon proteins are not submitted to selective constraints while transposing within a given host lineage, in turn explaining why their persistence over time relies more on HT than that of retrotransposons. And most notably, our results provide evidence that retrotransposons generally evolve under purifying selection in vertebrates, not only during horizontal transfer between organisms, but also within host lineages. This pattern posits *cis*-preference as a key selective filter allowing retrotransposons to persist over long periods of time under strict vertical transmission. Overall, these results contribute to the depiction of TEs as genomic symbionts that replicate, move between hosts lineages and diversify under natural selection, like symbiotic organisms do, while profoundly shaping the genome of their hosts.

## Methods

### Source data

We used 307 vertebrate genome sequences whose accession numbers are provided in Supplementary Data 1. These sequences constitute the set that was available on GenBank as of December 2017, excluding 13 species whose genome sequence was considered too short or of insufficient quality. We built a timetree of these taxa (Supplementary Data 5) using timetree.org^54^. Most of the following analyses were automated via custom scripts written in R^55^.

### Transposable element annotation and similarity search

The de novo annotation of TEs via RepeatModeler v. 1.0.10^30^ used the parameter “ncbi” as search engine to generate TE family consensus sequences. We excluded consensuses presenting non-TE genes^24^ by performing blastx searches of consensus sequences against the non-redundant database of proteins from NCBI. These searches used Diamond v. 0.9.19^56^. We also excluded TEs recognized as Short Interspersed Nuclear Elements (SINEs), which do not contain protein-coding regions required for Ka and Ks computations^24^. Remaining consensuses that were assigned to defined TE superfamilies (i.e., characterized below the level of TE subclass) were used to locate TE copies in each genome with the RepeatMasker program v. 4.0.7^31^, ignoring low complexity regions. Copies were extracted from contigs/scaffolds with seqtk v. 1.2-r94 (https://github.com/lh3/seqtk).

All blast searches used ncbi blast version 2.6.0+^32^. For every relevant pair of genomes, we performed reciprocal blastn searches between TE copies of at least 300 bp, reporting only the best alignment per query sequence per search^24^. We retained alignments of at least 300 bp in length, with sequence identity ≥75%, quality score ≥200 and between TEs assigned to the same superfamily. This last criterion was fulfilled by ~98% of the hits passing the three previous ones, indicating that the automatic TE classification was generally accurate.

### Estimating synonymous and non-synonymous TE mutation rates

Ka and Ks rates were computed on homologous regions of pairs of TE copies, for different stages of this analysis (see next subsections). This computation required delineating protein-coding regions in these TEs. This was achieved through successive similarity searches^24^ with Diamond^56^, of TEs copies against the database of TE proteins used by RepeatModeler. More than 99% of the blastx hits involved a TE and a protein assigned to the same superfamily, again supporting the accuracy of the TE classification. The remaining <1% of hits between a TE and a protein of different superfamilies were discarded. Homologous TE regions of a TE–TE hit, as reported by blastn, were extracted from TE copy sequences with seqtk and realigned using the Biostrings R package v. 2.52^57^. Every aligned base in each TE copy was attributed a position within a codon based on the Diamond blastx alignment coordinates of TE copies on proteins. Nucleotides of undetermined or mismatched within-codon positions between copies were deleted, so were indels and resulting truncated codons. On the remaining codons, Ka–Ks rates were computed with Li’s method^58^ implemented in the seqinr R package v. 3.4–5^59^. This procedure effectively reconstructs (partial) homologous protein-coding sequences from a pair of TEs.

### Selecting TE similarities resulting from HTT

Core genes were annotated from the genome sequences by the BUSCO v. 3.0.1 pipeline^60^, using the database of single-copy proteins that was the most relevant for each vertebrate group. Our aim was to generate core-gene Ks distributions for every pair of sister clades, as done in recent studies^24,25^. Each distribution therefore represents the degree of neutral molecular divergence between two lineages more accurately than a distribution based on a single species pair. This approach also allows using species that have few annotated genes (for instance, no core gene could be annotated for the collared flycatcher *Ficedula albicollis*). To reduce the workload, Ks distributions involving two sister clades older than 250 My used just one genome per subclade younger than 30 My, the one with the highest number of annotated core genes.

Pairwise Ks between gene orthologs of selected species pairs were computed using Li’s method^58^, as we did for TEs (see above). Each Ks distribution comprised Ks values from pairwise alignments of at least 600 bp, retaining only the longest alignment among those involving the same gene (considering orthologs from different species as different genes) to reduce pseudo-replication.

We computed Ka and Ks rates of homologous TEs on the TE–TE hits, as described in the previous subsection. To reduce the computational load, we first discarded any hit for which the global sequence identity (the percentage identity, or “pID” reported by blast) was higher than the 0.5% quantile of the core gene Ks distribution from the sister clades corresponding to the species pair involved in the hit. Such hit would have been unlikely to pass the following filter.

We removed any TE–TE hit for which the Ks value, added to twice the Ks standard deviation reported by seqinr, was higher than the 0.5% quantile of the Ks distribution of core genes from the sister clades corresponding to the pair of species involved in the hit. We also removed all hits whose Ks value was ≥0.5 or computed on less than 100 codons.

### Delineation of HTT events by clustering of hits between TEs

The computing resource and time required by our hit clustering approach led us to reduce the number of hits beforehand. We thus selected a subset of hits among each set that could already be inferred to result from the same HTT event. This inference was based on the fact that many hits share TE copies (e.g., a hit between copy A and copy B and a hit between copy A and copy C), and likely represent the same transfer. We applied single-linkage clustering^24^ to connect any two hits sharing a TE copy and we retained no more than 200 hits per resulting hit cluster per species pair. In this selection, we favored hits covering the longest protein-coding regions between TEs.

We then clustered retained hits into groups representing different HTT events, separately for each TE superfamily, in a two-iteration procedure. The first iteration was restricted to hits between the same two vertebrate clades younger than 40 My. Collapsing species into young clades, within which HTT was not searched, allowed applying the clustering procedure we previously developed^24^. According to this procedure, hits are inferred to result from the same HTT event (according to what we hereafter call “criterion 1”) if there is higher sequence identity within at least one clade than between clades, for the TE copies involved in the hits. Sequence identities between TE copies of the same clade were obtained through blastn searches of all copies of a superfamily (those in the retained hits) against themselves, without restriction on the number of results per query, but discarding alignments shorter than 100 bp.

Any two hits passing criterion 1 were “connected”, resulting in an undirected graph of hits, within which groups of hits (hereafter called “communities” to avoid confusion between iterations) were delineated by the clustering algorithm^61^ implemented in the igraph R package v. 1.2.4.1.

In the second iteration, we generated pairs of communities to evaluate if hits composing the two communities of a pair could result from a single HTT event, this time by applying both criterion 1 and our newly-developed criterion (“criterion 2”) that compares the degree of divergence of species to that of TE copies (Fig. 1).

Criterion 1 was considered satisfied if it was passed by ≥5% of all possible pairs of hits taken from the two communities^24^. If the sets of TE copies composing the two hit communities did not show any DNA sequence homology (no blast hit) and insufficient (<100 bp) protein sequence identity (as determined by blast searches of TE copies against a database of TE proteins^24^), we considered that these sets of copies could represent non-overlapping parts of a TE that underwent fragmentation or differential degradation within genomes after a unique transfer. In such case, we considered criterion 1 as passed. Criterion 2 is detailed in the Supplementary Methods and Supplementary Fig. 7. Briefly, its evaluation involved comparing the average Ks of hits composing the two communities, which represents the time since the transfer(s), to the 0.5% quantiles of Ks distribution of cores genes, which represents the time since divergence of the relevant host lineages.

Any two hit communities passing both criteria were consider as resulting from the same HTT event and were “connected”. On the resulting graph of communities, groups of communities (“hit groups”, as mentioned in the Results section and hereafter) were delineated through a complete-linkage clustering algorithm^24^. Complete linkage ensures that all communities within a hit group result from the same inferred HTT event. A hit group thus represents a direct or indirect HTT event between two clades.

### Hit group evaluation

We evaluated whether each hit group comprised a sufficient number of TE copies, to reduce the risk of considering between-genome DNA contamination as HTT. Since the scale of our analysis required discarding many hits and associated TEs, we aimed to retrieve TEs copies for each hit group. We did so through blastn searches of TE copies constituting hit groups against all ≥100-bp-long copies from their host species, discarding hits shorter than 100 bp. For a “retrieved” TE copy to be attributed to a clade (called “clade A”) involved in a HTT (i.e., a hit group) between clades A and clade B, the average sequence identity (reported by blastn) between this copy and clade-A copies of the hit group must be higher than the average sequence identity between clade-A copies and clade-B copies (given by the pID of hits within the hit group). We discarded each hit group that comprised less than five TE copies per clade, including retrieved copies, or less than two copies per clade, not including retrieved copies.

We further assessed whether the Ks distribution of hits within each group was visibly truncated to the right by our filter involving Ks thresholds. In such case, retained hits in a group might represent pairs of TE copies that happened to have diverged much more slowly than average after a speciation event, i.e., the Ks of the hit group might constitute the left tail of a larger Ks distribution that represents vertical inheritance rather than HTT^18^. To account for this risk, any hit group was removed if the modal class of its Ks distribution did not contain at least 20 more hits than the rightmost class, or if its maximum Ks was higher than the Ks threshold used to select hits, minus 0.2. The Ks threshold is the smallest value between 0.5 and the 0.5% quantile of the Ks of core orthologous genes from the corresponding clades (see subsection “selecting TE similarities resulting from HTT”). Ks classes were delineated by the hist() function of R.

### Counting independent HTT events

We counted independent HTT events through a procedure that analyzes the retained hit groups successively. This procedure is illustrated in Supplementary Fig. 8 and represented as pseudocode in the Supplementary Methods. This procedure evaluates whether a given TE copy we call “copy A” from the currently processed hit group (hereafter called the “focal transfer” between clade A and clade B) could have been brought into its host species (belonging to clade A) through a transfer corresponding to another hit group (called “explanatory transfer”). This requires (i) that the explanatory transfer involves a vertebrate clade (called “clade C”) that is the same as, nested in, or encompassing clade A, and (ii) that copy A has higher sequence similarity to a copy from clade C in the explanatory transfer than it has with at least one copy from clade B in the focal transfer. These conditions are evaluated for every copy involved in the focal transfer, considering all possible explanatory transfers. We call “requirement 1” the fulfillment of these two conditions for at least two different explanatory transfers and for at least one TE copy in every species involved in the focal transfer. If ignoring any explanatory transfer prevents requirement 1 from being fulfilled, this explanatory transfer is flagged as “required” to explain a focal transfer. To be considered as “explained”, a focal transfer must comply with requirement 1 and must not be required (to explain any previously processed hit group). It is then removed from the pool of possible explanatory transfers.

Focal transfers are processed in order of increasing “reliability” score, starting with hit groups that are more likely to constitute indirect transfers. This reliability score is computed by obtaining the highest blast pID among hits involving each TE copy, summing these pIDs over copies from either clade of the hit group, and taking the lower of the two sums. Processing hit groups in the reverse order (hit groups with highest scores first) only slightly increased (by ~10%) the minimal estimated number of HTT events.

The 975 hit groups that could not be fully explained by others at the end of this procedure were considered as representing as many independent HTT events and were used in the following analyses. We henceforth simply refer to these hit groups as “transfers”.

### Analysis of HTT distribution across vertebrate clades

We devised a species-permutation procedure to evaluate the deviation of the HTT distribution from a null distribution, separately for each TE superfamily except for those involved in less than 20 transfers, which we combined in each TE class. These permutations required assuming that each of the 975 retained transfers involved only two species. We chose the species pair constituting the hit of highest sequence identity in each transfer. Should a transfer have involved an ancestor of several studied species, this selection can be seen as a random choice between descendant species^24^.

A permutation shuffled species among those we associated with the transfers of a given TE superfamily. A given species is therefore replaced by the same species across all transfers where the former species is involved. Any permutation leading to at least one transfer between species that diverged in the last 120 My was discarded as illegal. We repeated this procedure until 1000 legal permutations were obtained. For TE superfamilies involved in more than 120 transfers, obtaining 1000 legal permutations proved unachievable in a reasonable timeframe, a problem faced by other authors^25^. We therefore performed permutations on arbitrarily delineated subsets of transfers within these superfamilies, at the cost of a slight decrease in statistical power.

The effect of habitat (terrestrial or aquatic) on HTT distribution was tested by permutating habitats of tetrapod species involved in transfers with ray-finned fishes. As these permutations cannot be illegal, but have limited statistical power due to the scarcity of aquatic tetrapods, we performed this analysis on all 2632 hit groups (not just the 975 independent transfers) involving tetrapods and ray-finned fishes, pooling all TE superfamilies.

### Estimating rates of molecular evolution by transposition

To estimate Ka and Ks substitution rates between TEs that diverged through transposition only, we used pairs of homologous TEs from the same hit “community” (see subsection “Delineation of HTT events by clustering of hits between TEs”) and genome. These TEs are unlikely to have undergone HTT during their divergence. For this analysis, we selected pairs of TE copies that were aligned over ≥300 bp of protein-coding regions, based on the results of the blastn searches we conducted to evaluate criterion 1 (see subsection “Delineation of HTT events by clustering of hits between TEs”). Ka and Ks rates were computed as described in subsection “Estimating synonymous and non-synonymous TE mutation rates”

### Reporting summary

Further information on research design is available in the Nature Research Reporting Summary linked to this article.

## Supplementary information

Supplementary Information

Peer Review

Reporting Summary

Description of Additional Supplementary Files

Supplementary Data 1

Supplementary Data 2

Supplementary Data 3

Supplementary Data 4

Supplementary Data 5

## Data Availability

Data supporting the findings of this work are available within the paper and its Supplementary Information files. Accessions for all genomic data analyzed in this study are listed in Supplementary Data 1.

## Code availability

All code used to perform the analysis, including the generation of result-based figures and tables, is available on GitHub at https://github.com/jeanlain/HTvertebrates.
