# Supplementary material for: Horizontal transfer and evolution of transposable elements in vertebrates
Source: Nat Commun. 2020 Mar 13;11:1362. doi: 10.1038/s41467-020-15149-4 (PMC7070016; doi:10.1038/s41467-020-15149-4)
Supplement: Supplementary file 1 — Supplementary Information [file 41467_2020_15149_MOESM1_ESM.pdf]

Supplementary Information for

Horizontal transfer and evolution of transposable elements in vertebrates

Zhang et al.

This PDF file includes:

Supplementary Discussion 2

Lower number of HTT involving mammals compared to previously published results 2

Impact of the pairwise approach on the reliability of the Ka/Ks analysis 2

Sensitivity of Ka/Ks to selection acting on transposable elements 4

Supplementary Methods 4

Detailed two-step clustering procedure and application of "criterion 2" 4

Pseudocode representation of the procedure that counts independent HTT events 5

Supplementary Figure 1 8

Supplementary Figure 2 9

Supplementary Figure 3 10

Supplementary Figure 4 11

Supplementary Figure 5 12

Supplementary Figure 6 13

Supplementary Figure 7 14

Supplementary Figure 8 15

Supplementary Table 1 16

Supplementary Table 2 17

Supplementary references 18

Supplementary Discussion

Lower number of HTT involving mammals compared to previously published results

We inferred that only one independent horizontal transfer of RTE-BovB elements involved mammals and squamates. This event is represented by 7680 hits that constitute hit group #6254 in Supplementary Dataset 4.

By contrast, Ivancevic et al.[1] detected many transfers of BovB elements between mammals and squamates. Depending on the criteria used to count independent transfers (A. M. Ivancevic, personal communication), 12 to ~50 independent transfers of BovB involving mammals and squamates can be delineated. The much lower number of HTT events we detected may be explained by critical differences between the approach of Ivancevic et al. and ours.

First, Ivancevic et al. detected homologies between TEs at the protein sequence level (in addition to the DNA sequence level), which permits the detection of potentially much older HTT events. By contrast we excluded any hit involving TEs whose DNA sequence homology was <75% and whose Ks was <0.5 (see method section " Selecting TE similarities resulting from HTT").

Second, we applied a very stringent filter that compared the Ks between TEs to that of core genes. In particular, we excluded any hit groups if the distribution of Ks of the hits appears truncated before reaching its mode (see method section "Hit group evaluation"). If we remove this "truncation" criterion, 16 hit groups of BovB elements involve mammals and squamates.

Third, our criterion to reduce the risk of DNA contamination being taken as HTT is based on the number of copies detected in genomes, whereas that of Ivancevic et al. is based on comparisons of TE copy flanking regions. Our criterion might have excluded transfer events that did not amount to contamination. Without applying this criterion, the number of hit groups of BovB elements involving mammals and squamates increases to 25.

Finally, our very aggressive clustering procedure may have placed TE-TE hits resulting from different transfers into the same hit group, as we considered TEs copies sharing no homology in their nucleotide and protein sequences as possibly representing non-overlapping parts of the same TE (see method section "Delineation of HTT events by clustering of hits between TE copies"). Hence, independent transfers of BovB elements that do not show detectable homology may be considered as a single transfer, but would constitute different clusters in the approach used by Ivancevic et al.

Impact of the pairwise approach on the reliability of the Ka/Ks analysis

As opposed to substitutions rates obtained from a tree-based approach, those measured by our pairwise method cannot be estimated during specific parts of a gene's evolutionary history (i.e., along specific tree branches), nor can they be obtained with advanced, realistic models of nucleotide sequence evolution. We evaluate how these limitations might affect the reliability of our analysis of Ka/Ks ratios.

First, the mutational model we used may underestimate substitution rates if several substitutions occurred at the same sites, a common limitation of basic nucleotide substitution models. Under a regime of purifying selection, recurring mutations are more likely to be synonymous, leading to an overestimate of the Ka/Ks ratio due to saturation of Ks, hence to false inference of relaxed selection.

To investigate this issue, we plotted Ks as a function of Ka (Supplementary Fig. 3), separately for TEs that diverged via HTT and those that did not, and for the different TE categories that we discuss in the main text (section "Selective constraints acting on transposable elements"). Saturation of Ks results in plateaus, as most visible in LINE elements involved in horizontal

transfer (Supplementary Fig. 3, bottom-left plot, red dots). Before plateaus however, the slopes of the Ka/Ks relationship clearly differ depending on the occurrence of HTT (i.e., slopes differ between red and black dots on each plot). The effect of horizonal transfer on Ka/Ks is therefore not due to Ks saturation. The slopes also differ between TE types, for TEs that diversified within genomes (black dots). Hence, higher Ka/Ks ratio for Jockey and DNA transposons that diversified within genomes cannot result from Ks saturation.

The effect of Ks saturation may be confounded with another factor that our method cannot account for, which is TE deactivation. At least one of the homologous TE copies of a pair may be deactivated by a mutation during part of their divergence. Subsequent mutations on this copy should all be neutral, hence move the Ka/Ks ratio toward unity, even though the initial divergence between the TEs occurred under some form of selection.
To assess how the effect of TE deactivation might be confounded with the ones we study (TE type and the occurrence of HT during TE divergence), we reasoned that the overestimate of Ka/Ks due to these factors should increase with the degree of TE sequence divergence. Indeed, sequence divergence should correlate with the time and frequency mutations after TE deactivation. We thus plotted (Supplementary Fig. 3) a metric that contrasts the Ka and Ks rates measured on pairs of copies against their degree of sequence divergence. The metric we used is (Ka – Ks)/(Ka + Ks) instead of Ka/Ks because averages of Ka/Ks ratio are expected to exceed unity under neutral evolution, simply due to right-skewness of Ka/Ks distributions (e.g., Fig. 4B, main text). Averaged Ka/Ks ratios exceeding unity may give the false impression of positive selection. (Ka – Ks)/(Ka + Ks) varies from –1 to 1 and its distribution is relatively symmetrical around zero. A value of zero reflects neutral molecular evolution (Ka = Ks) and a negative value is expected under purifying selection. This metric responds to selection in the same direction as Ka/Ks does. The overall sequence divergence between copies was measured at all aligned sites (ignoring indels) using the mutational model[2] underlying the method we used[3] to estimates Ka and Ks.
Supplementary Fig. 4 shows that our metric does not increase with overall sequence divergence between TEs and shows no evidence that the variations of Ka/Ks ratios that we interpret in terms of selection regimes are actually due to differences in the degree of TE degradation. Our metric indeed varies among TE categories that display largely overlapping ranges in pairwise TE sequence divergence, and it is consistently lower for TEs that diverged though HTT than for TEs that did not, at the same degree of sequence divergence.

A third factor that is not taken into account by our approach is non-stationarity in DNA base composition during sequence evolution, which may bias estimates of Ka/Ks. This bias may amount to an underestimate or to an overestimate of Ka/Ks, depending on how G+C composition evolves [4, 5]. How G+C composition of a locus evolves may depend on the G+C composition of its genomic context. To evaluate if variation in G+C content could be confounded with the factors we investigate, we plotted (Ka – Ks)/(Ka + Ks) against the difference in G+C proportion between the contigs carrying the divergent copies (Supplementary Fig. 5). We also plotted our metric against the difference in G+C proportions of the TE copies themselves (Supplementary Fig. 6). In either case, the effect of G+C composition on (Ka – Ks)/(Ka + Ks) is negligible in comparison to the effect of TE divergence mode and TE category.

In sum, these uncontrolled factors cannot explain the patterns of Ka/Ks that we interpret in terms of natural selection (main text), mainly the Ka/Ks ratios that are lower than one for most TEs

except Jockey and DNA elements that diverged within genomes. We however advise against using the Ka and Ks values reported in Supplementary Dataset 4 in studies that requires highly accurate estimates.

Sensitivity of Ka/Ks to selection acting on transposable elements

The selection regime that we investigated by measuring Ka/Ks ratios between different TE copies is largely independent of the hosts' fitness. In that regard, whether a particular mutation in a TE copy has fixed in a host species is irrelevant.

Removing the host species from the picture, the diversification of a TE within a genome can be assimilated to that of a population of clonal organisms. Mutation that differentiate TEs copies of a family are not fixed from a TE population viewpoint, like alleles of a polymorphic locus. Mutation that reduce a TE's ability to transpose may not be purged from the host species, hence may be present. Such mutations are however less likely to be replicated by transposition, hence, to appear in several TE copies. These deleterious mutations would therefore become rarer and eventually disappear as non-replicating TEs are degraded/deleted.

Kryazhimskiy and Plotkin [6] have shown that Ka/Ks ratios measured between variants/clones of the same population are less sensitive to selection than those traditionally calculated from mutations that are fixed between species. It is likely the case for mutations that differentiate TE copies. Ka/Ks that do not significantly differ from unity may therefore not be taken as evidence for purely neutral evolution of TE protein-coding sequences.

However Ka/Ks ratios lower than one are still indicative of negative (purifying) selection. Indeed, Ka/Ks ratios measured within populations decrease with the strength of negative selection, like they do when measured between species (the decrease is only less abrupt, see Fig. 1 of Kryazhimskiy and Plotkin [6]).

Supplementary Methods

Detailed two-step clustering procedure and application of "criterion 2"

The first clustering iteration (see method section "Delineation of HTT events by clustering of hits between TE copies") processes only groups of hits between two young clades, the most recent common ancestor (MRCA) of each being less than 40 My old. Application of criterion 1 resulted in “communities” of hits, each representing a HTT event between these two clades.

Two communities of hits formed by this first iteration of clustering may not constitute two separate HTT events, under different scenarios illustrated by Supplementary Fig. 7: (i) a single TE might have been transferred into a common ancestor of two clades (clades C and D); (ii) the “donor” clades (clades A and B) may share similar TEs through vertical inheritance. In the latter, the transfer need not even involve a common ancestor of clades A and B (Supplementary Fig. 7) to cause TE homology between clade A and clades C-D.

These scenarios can only apply to communities of hits that involve clades that share the same MRCA. This is the case for every pair of hit communities shown on Supplementary Fig. 7. For instance, clade pair A-D (for hit community AD) and clade pair B-C (for hit community BC) have the same MRCA, which is the basal node of tree. Hits between TEs from clades A and B and between TEs from clades C and D (not shown) cannot results from the same transfer event. Likewise, communities AC and CD cannot result from the same transfer, since community CD (not shown) must represent a HTT between clades C and D, which cannot be the same event as a HTT involving clade A or B.

To evaluate these scenarios, we compare synonymous divergence between TEs composing communities, to synonymous divergence between species. For the former, we use the mean Ks of hits within each community. For the latter, we use the 0.5% quantiles of the Ks distributions of core genes from the two pairs of most closely related clades among those involved. In the case show on Supplementary Fig. 7, these clade pairs are A-B and C-D. The Ks distributions or core genes corresponding to these clade pairs' MRCAs (tree nodes at t1 and t2 on Supplementary Fig. 7) are retrieved from those we established. If two communities have a clade in common (for instance, communities AD and BD), one clade pair is actually a single clade (DD). For this clade pair, the Ks of core genes is set to 0. We used a quantile-based approach rather than statistically comparing the Ks of hits within communities to that of core genes, as Ks values within a community (a TE tree) are not independent, violating conditions of a two-sample test.

The two communities will pass criterion 2, and will be considered to possibly represent the same transfer, if the following two conditions are met:

- The mean Ks values of the two communities must both exceed the lowest of the two 0.5% Ks quantiles of the clade pairs. This is the case of every pair of communities on Supplementary Fig. 7. Should a hit community (not shown on the figure) present a Ks that is lower than the synonymous divergence between clades C and D, the corresponding transfer could not have occurred in a common ancestor of clades C and D.
- The mean Ks of least one community must be at least as high as the highest of the 0.5% Ks quantiles of the two clade pairs. This is the case for every pair of communities on Supplementary Fig. 7, since the divergence of TEs from communities AD or AC should correspond to the divergence between clades A and B. Should hit community AD (or AC) present a Ks that is lower than t1, the homology between TEs of clades A and D (or clades A and C) could not possibly be due to a transfer involving an ancestor or a member of clade B. An ancestor/member of clade A must have been involved in a transfer.

A pair of communities failing to meet either condition will not pass criterion 2.

Since our first iteration of clustering confronts hits between the same pair of young clades (for instance clades A and C on Supplementary Fig. 7), criterion 2 was not applied at this stage. Indeed, the Ks of species within clades younger than 40 My was not computed, and considered to be 0. The two conditions of criterion 2 are necessarily met.
In principle, criterion 2 can apply to a pair of hits rather than a pair of hit communities. However, as this criterion uses Ks thresholds, two hits resulting from the same HTT might have Ks values falling at opposite sides of the 0.5% quantile of the Ks of core gene orthologs. These two hits may therefore end up into different communities. While this issue can still happen for two hit communities, we believe that our very aggressive clustering (criterion 2 compares mean Ks between TEs to 0.5% quantiles of Ks between gene orthologs) makes it unlikely for this issue to yield an excess of inferred HTT events.

Pseudocode representation of the procedure that counts independent HTT events

In the pseudocode below, the colors of certain variable names (blue or red) correspond to elements shown on Supplementary Fig. 8.
Variable/object names are italicized, and common operations are written in bold.
Comments that are not part of the pseudocode are preceded with by: #.

We **define** as a *transfer* a group of *hits* (a hit group as defined in the main text) between *TE copies* from *species* belonging to two *clades* (see Supplementary Fig. 8)

We **define** *explanatory_transfers* as a list initially containing all *transfers*

We **define** the *lowest similarity* of a *TE_copy* in a *transfer* as the lowest sequence similarity across all *hits* that involve this *TE_copy* among *hits* of the *transfer*

Below, we define a function that evaluates whether a transfer can be explained by others, i.e., if
"requirement 1" (as defined in the method section "Counting independent HTT events") is
passed.

Function *requirement* is **applied to** a *transfer* called *focal_transfer*:

We **initialize** *explained_species* and *contributing_transfers* as empty lists

for each *TE_copy* **among** the TE copies involved in the *focal_transfer* we **do:**

We **define** *species_A* as the species to which the *TE_copy* belongs
We **define** *clade_A* as the clade to which the *species_A* belongs, among the two *clades* involved in the *focal_transfer*

for each *explanatory_transfer* **among** the *explanatory_transfers*, **excluding** the *focal_transfer*, we **do:**

We **define** *highest_similarity* as the highest sequence similarity between the *TE_copy* and any TE copy from the *explanatory_transfer*

if either *clade* involved in the *explanatory_transfer* is nested in, **or** encompasses, *clade_A* **and if** the *highest_similarity* is **higher than** the *lowest_similarity* of the *TE_copy* in the *focal_transfer* **then:**
We **add** *species_A* **to** the list of *explained_species*
We **add** the *explanatory_transfer* **to** the list of *contributing_transfers*
end if

end for

end for

if the *explained_species* **contain** all the *species* involved in the *focal_transfer*
and the *contributing_transfers* **contain at least** two different *transfers* **then:**
We **return** PASSED
else:
We **return** NOT_PASSED
end if

end function

We inspect transfers iteratively to evaluate if they can be “explained” by others.

We **initialize** *explained_transfers* as an empty list

for each *focal_transfer* among all *transfers* sorted by increasing reliability (see methods) we **do:**

  if *requirement_1* **is** PASSED for the *focal_transfer* **then:**
    We **remove** the *focal_transfer* **from** the list of *explanatory_transfers*
    if *requirement_1* **is** NOT_PASSED **for any** *transfer* **among** the *explained_transfers* **then:**
      We **restore** the *focal_transfer* **into** the list of *explanatory_transfers*
    else:
      We **add** the *focal_transfer* to the list of *explained_transfers*
    end if
  end if

end for

The transfers that are not in the list of *explained_transfers* are those considered as
“independent” in the main text.

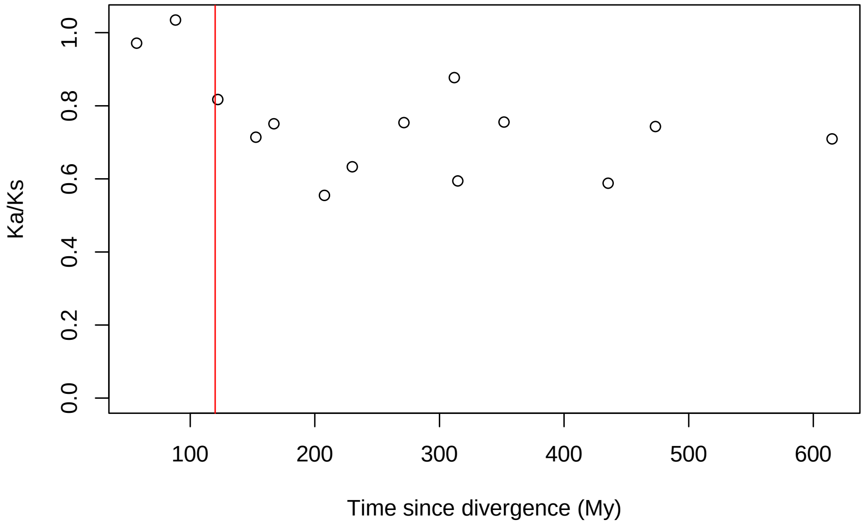

Supplementary Figure 1

Correlation between the time since divergence of vertebrate genomes sharing homologous TEs and the Ka/Ks ratios of these TEs, for DNA transposons involved in hits that passed filters designed to select homologies resulting from HTT. Each point represents the average of Ka/Ks ratios of many (~52 000) hits within a class of divergence time. Ka/Ks ratios are generally lower than 1, as expected if solely TE copies encoding functional proteins can horizontally transfer. However, the two classes of lowest divergence time show the highest Ka/Ks ratios, which are close to 1. These ratios indicate relaxed purifying selection, as expected for DNA transposons that do not undergo HTT. This pattern suggests that TE homologies between related lineages partly result from vertical transmission of TEs. It is expected that detected vertical transmission of TEs mostly involves closely related lineages, since homology between highly distance lineages is harder to detect. The vertical red line at 120 My indicates the time of species divergence below which we did not retain TE-TE hits, hence below which HTT was not investigated.

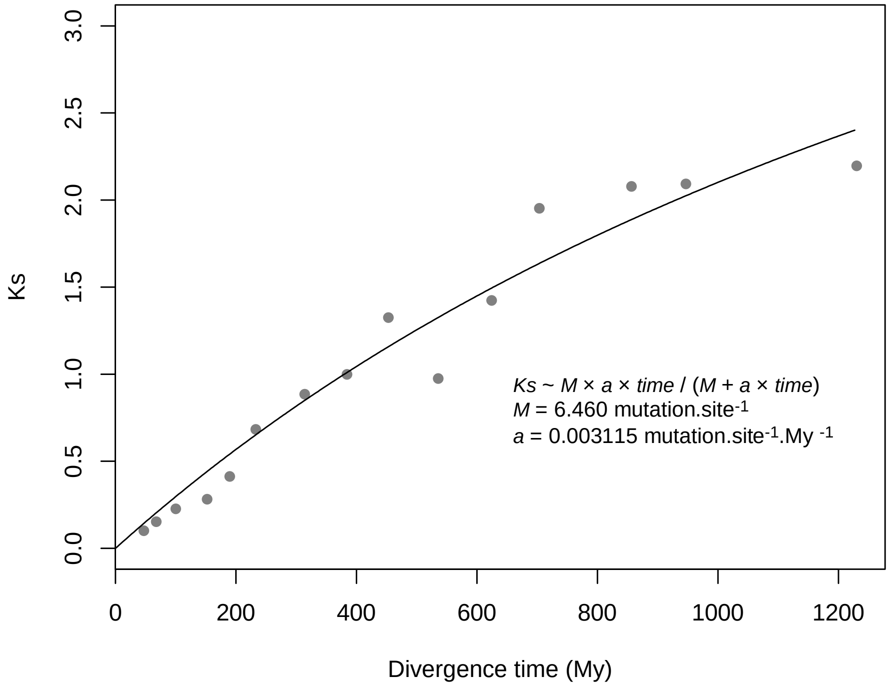

Supplementary Figure 2

Correlation between the time since divergence of vertebrate lineages and the synonymous mutation rate (Ks) established at core gene orthologs. Each point represents the average of many (~69 000) Ks values within a class of divergence time. The fitted curve assumes initial linear relationship between Ks and time (coefficient *a*) and a maximal value of Ks (*M*) due to mutational saturation. Divergence time is twice the time since the last common ancestor, to account for mutation in both diverging lineages.

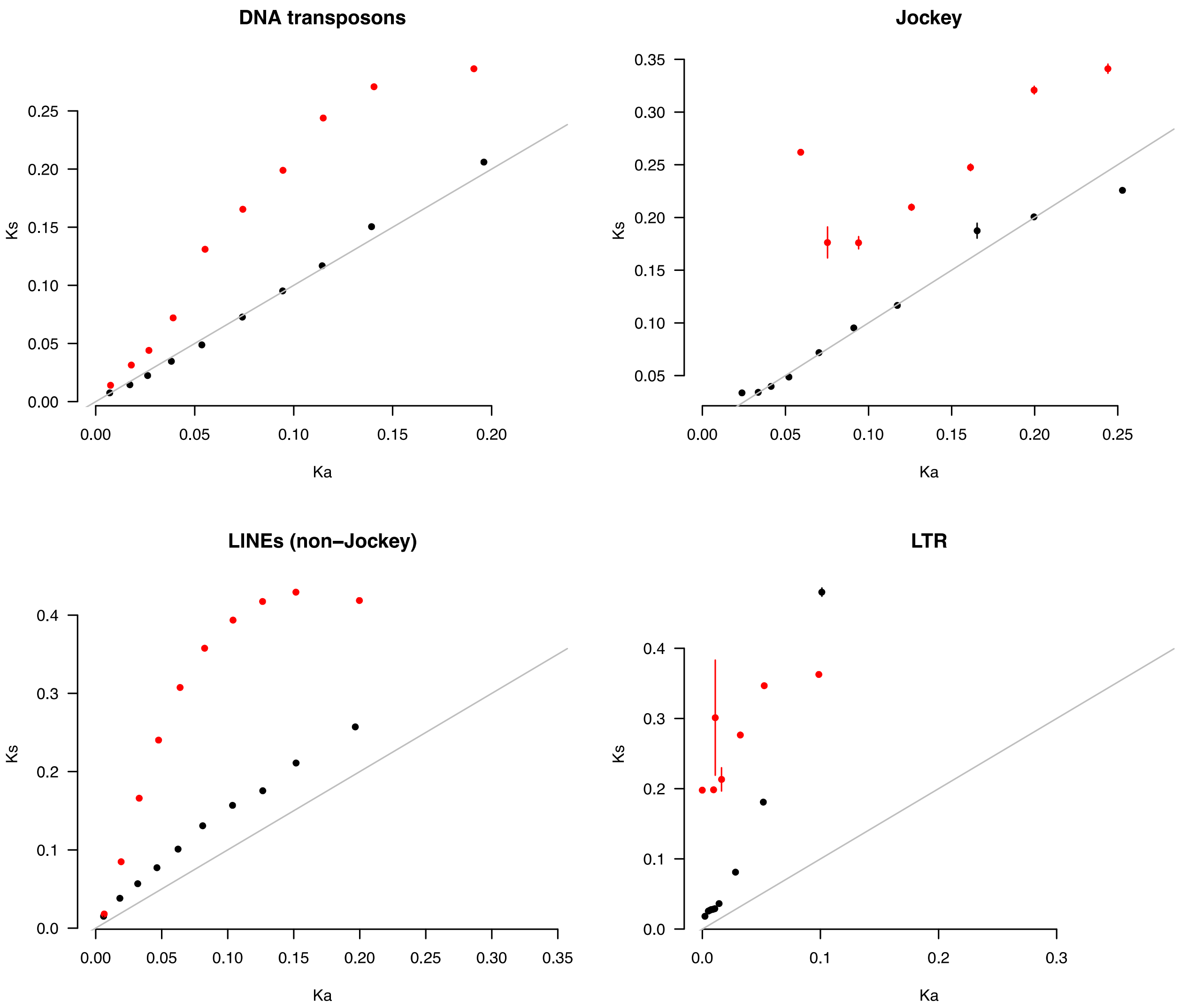

Supplementary Figure 3

Ks (synonymous substitution rate) as a function of Ka (non-synonymous substitution rate) measured on pairs of homologous TEs from the four TE categories that are outlined in the main text. For each plot, ten classes of Ka were defined to comprise the same number of TE pairs (several thousands). Points represent means over these pairs (± standard errors of means shown as vertical segments that are generally shorter than the point radii). Black points represent TE pairs that diverged within genomes and red points those that diverged partly through horizontal transfer. Certain classes may not comprise TE pairs that diverged via HT, hence the lower numbers of red points. Grey lines represent neutral evolution (Ka = Ks). Plot aspect ratios were uniformed by adjusting the range of X axes, to allow visual comparison of slopes between plots.

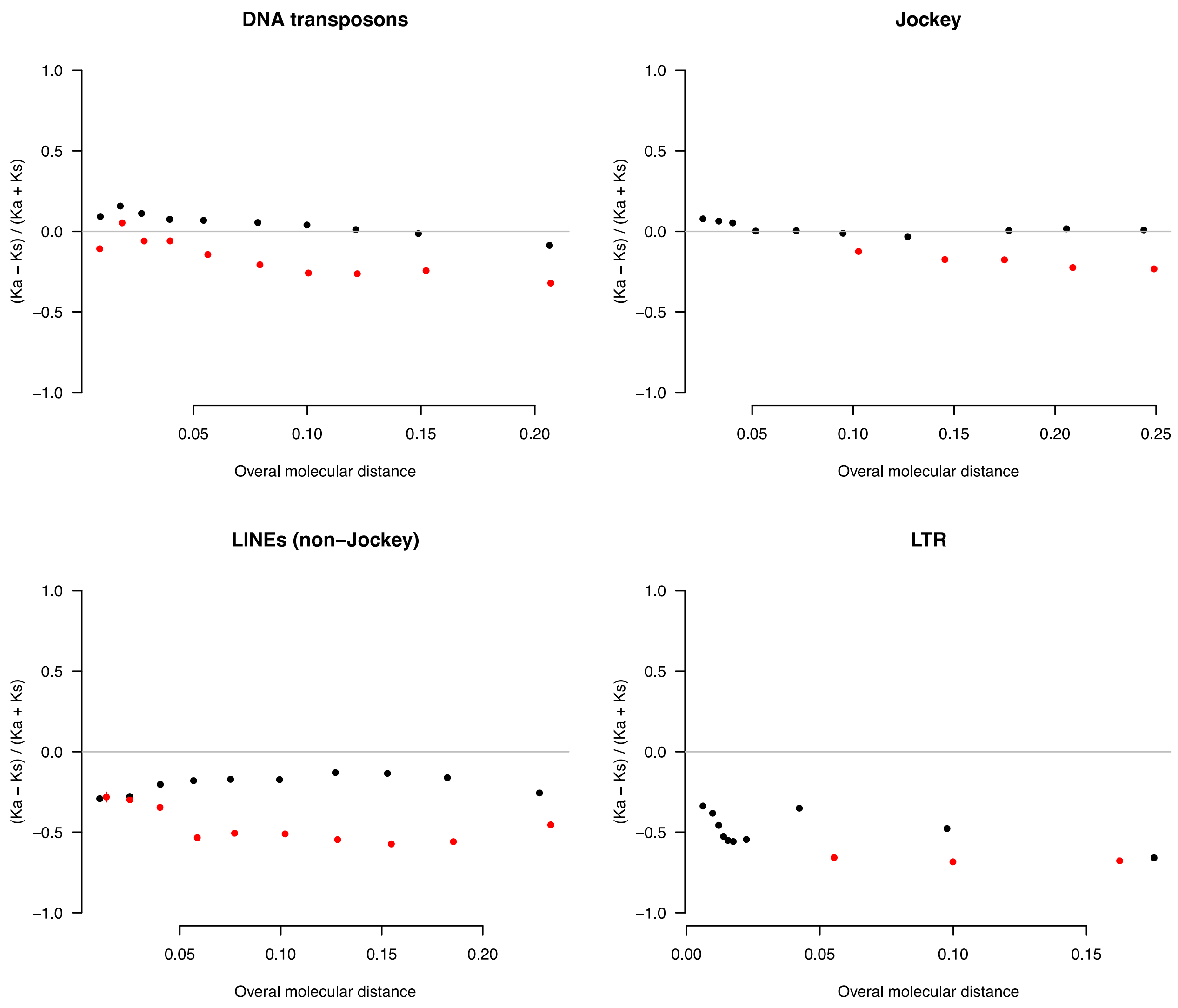

Supplementary Figure 4

A metric quantifying the selection regime acting on TEs (see Supplementary Text) as a function of overall molecular distance (all nucleotides considered), both measured on pairs of homologous TEs from the four TE categories that are outlined in the main text. For each plot, ten classes of the X-axis variable were defined to comprise the same number of TE pairs (several thousands). Points represent means over these pairs (± standard errors of means shown as vertical segments that are generally shorter than the point radii). Black points represent TE pairs that diverged within genomes and red points those that diverged partly through horizontal transfer. Certain classes may not comprise TE pairs that diverged via HT, hence the lower numbers of red points. Grey lines represent neutral evolution (Ka = Ks).

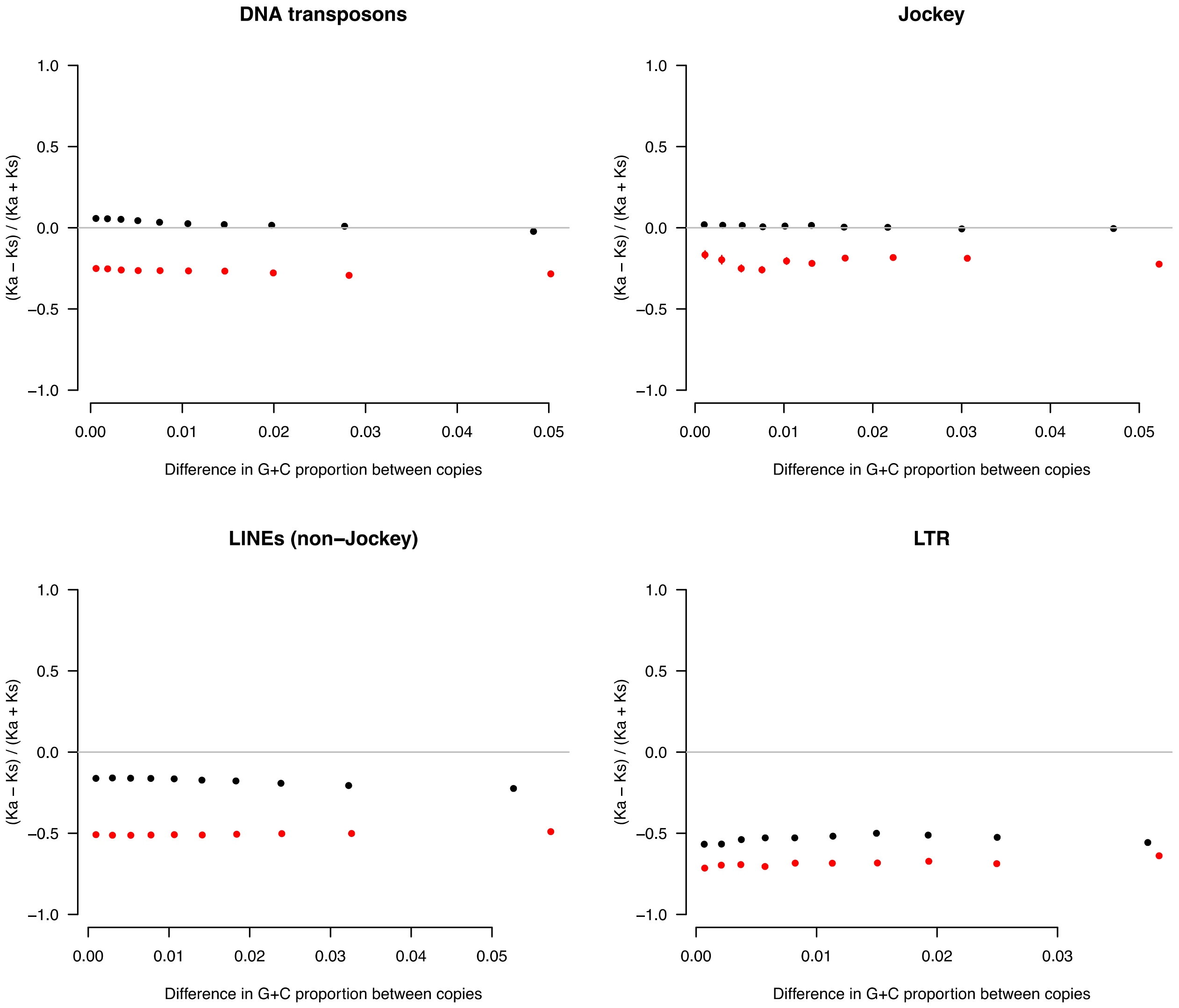

Supplementary Figure 5

A metric contrasting Ka and Ks (see Supplementary Text) as a function of the difference in G+C proportion, both measured on pairs of homologous TEs from the four TE categories that are outlined in the main text. For each plot, ten classes of the X-axis variable were defined to comprise the same number of TE pairs (several thousands). Points represent means over these pairs (± standard errors of means shown as vertical segments that are generally shorter than the point radii). Black points represent TE pairs that diverged within genomes and red points those that diverged partly through horizontal transfer.

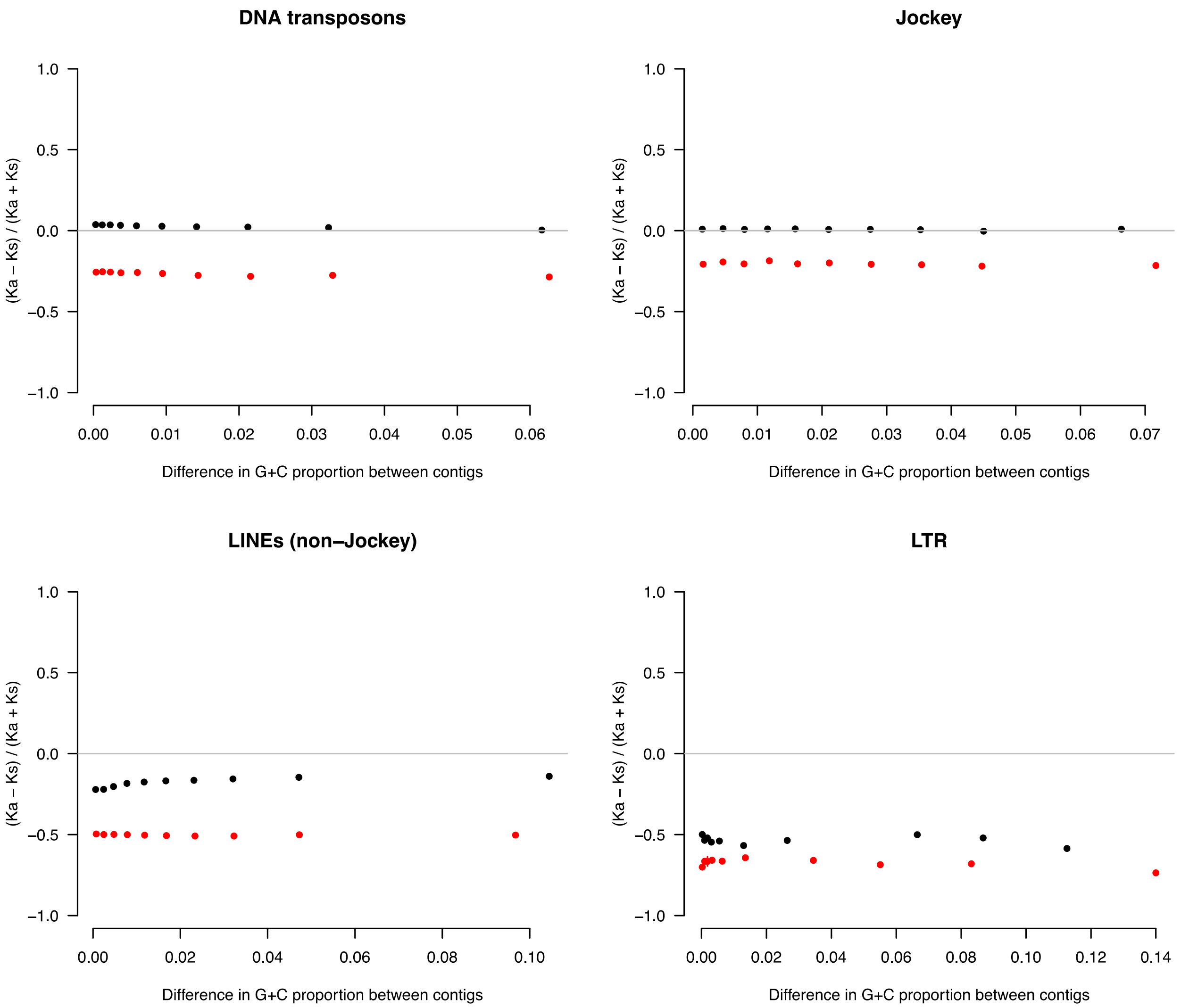

Supplementary Figure 6

A metric contrasting Ka and Ks (see Supplementary Text) as a function of the difference in G+C proportion between the host contigs, both measured on pairs of homologous TEs from the four TE categories that are outlined in the main text. For each plot, ten classes of the X-axis variable were defined to comprise the same number of TE pairs (several thousands). Points represent means over these pairs (± standard errors of means shown as vertical segments that are generally shorter than the point radii). Black points represent TE pairs that diverged within genomes and red points those that diverged partly through horizontal transfer.

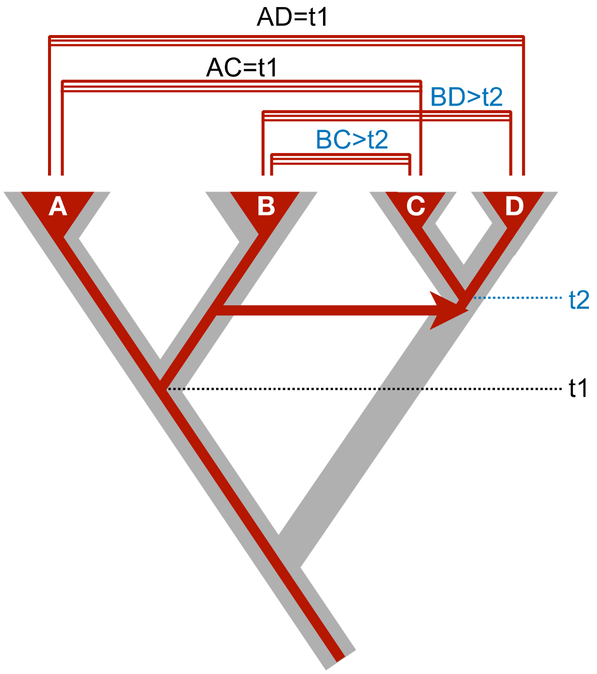

Supplementary Figure 7

A single HTT event leads to different communities of hits involving four young clades named from A to D, according to scenarios detailed in the SI Materials and Methods. The species tree is drawn with thick grey branches, and the tree of the TEs involved in the communities is drawn with red branches, the HTT event being represented by the arrow. t1 and t2 represent the divergence between clades A and B and between clades C and D, respectively. Hit communities are shown above the tree tips, each being named after the two clades it involves. "BD>t2" denotes the hits compositing community BD involve TEs that have diverged before t2.

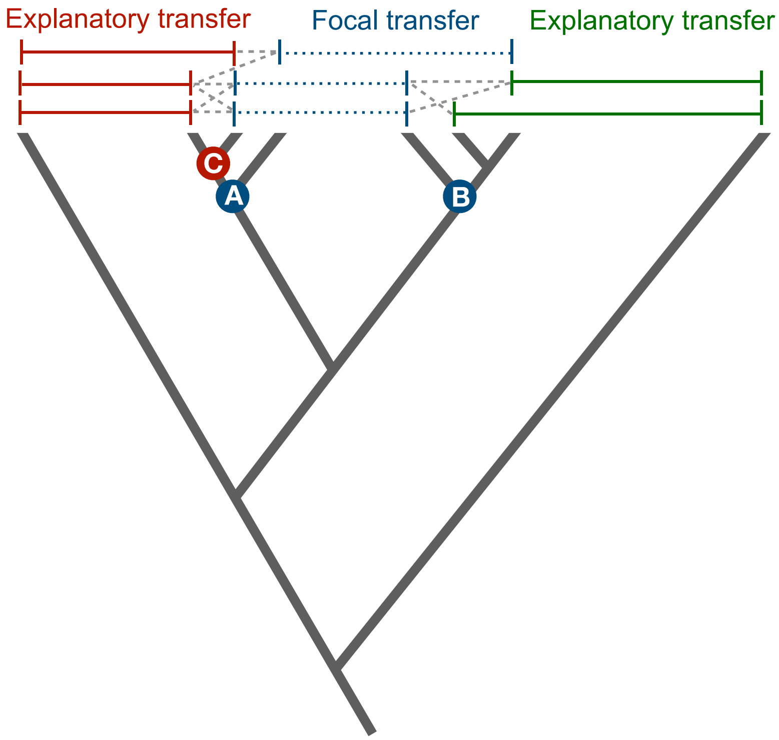

Supplementary Figure 8

Inferring whether a "focal transfer" of transposable elements (TEs) between two vertebrate clades A and B can be explained by other transfers ("explanatory transfers" shown in red and green). Transfers are shown as hit groups, a hit being represented by a horizontal segment connecting two short vertical segments representing TE copies. A TE copy belongs to the species that sits at the tree tip below it. The inference procedure is detailed in the Methods section. In this example, the left-hand explanatory transfer involves species composing clade C, which his nested within clade A, fulfilling one of the requirements to explain the focal transfer. A grey dashed line represents some degree of sequence identity between TEs involved in different transfers. This degree of identity must be lower than the identity in the hit of the focal transfer (represented by dotted lines) for at least one TE copy, in order to consider a transfer as "explanatory". Two explanatory transfers are required to consider the focal transfer as explained and to discard it from the count of independent transfer events.

Supplementary Table 1

Comparison between simulated and observed numbers of HTT events involving ray-finned fishes for different superfamilies of TEs.

**Superfamily**	**Mean over permutations**	**Highest among permutations**†	**Observed**†
Tc1/Mariner	150	249	388.5
hAT	10.20	19.5	20.5
other DNA*	4.9	10	10.5
Gypsy	10.20	23.5	29
Rex-Babar	61.42	103.5	165
LINE 2	45.09	88.5	121
R2-Hero	7.49	15.5	22.5
RTE	18.79	32.5	39
Other RNA*	22.03	36	49.5

* “Other” categories regroup families involved in less than 20 transfers. †If a given transfer involves a ray-finned fish and another vertebrate, 0.5 transfer is counted for ray-finned fishes, hence the decimal numbers.

Supplementary Table 2

Tests of the hypothesis that Ka/Ks ratios are lower than 1 for different superfamilies of vertebrate transposable elements that diverged through transposition only (upper half), or which underwent horizontal transfer during their divergence (lower half).

**Superfamily**	**Sample size***	**Mean Ka/Ks†**	**Mann-Whitney *U***	***p* value**
Tc1/Mariner	2663	1.2885	2951780	1
hAT	110	1.1240	4156	0.999503428
Other DNA	46	1.0838	711	0.969349297
Gypsy	48	0.6430	121	9.64E-08
Pao	26	0.5984	17	3.08E-06
Rex-Babar	833	0.7074	29398	4.01E-96
LINE 2	203	0.7714	3350	3.24E-17
RTE	569	0.9019	34848	2.35E-32
R2-Hero	32	0.8417	127	0.004669797
LINE 1	24	0.7812	56	0.002948821
Jockey	28	0.9906	187	0.364082817
Other RNA	69	0.8869	575	7.88E-05
Tc1/Mariner	363	0.6844	6478	1.66E-40
hAT	31	0.5113	10	2.00E-08
Other DNA	15	0.6520	17	0.006225586
Gypsy	29	0.1971	0	1.86E-09
Pao	17	0.2427	0	7.63E-06
Rex-Babar	169	0.3526	1	8.96E-30
LINE 2	120	0.4152	33	2.28E-21
RTE	52	0.4214	0	1.80E-10
R2-Hero	23	0.3482	0	1.19E-07
LINE 1	14	0.3495	0	6.10E-05
Jockey	10	0.6718	0	0.000976563
Other RNA	22	0.4307	1	4.77E-07

*Sample sizes correspond to the numbers of hit groups (or hit communities for the lower half of the table, see Methods), as each test used one value per hit group. This value is the mean Ka/Ks ratio over hits (TE-TE alignments) of the group, weighted by the length of alignments. We used only one value per hit group because pairwise distances among a set of objects are not independent. †The mean Ka/Ks is the average over these weighted means, not over the original hits' Ks values. Tests are one-sided and no ajustments for multiple tests were applied.

Supplementary references

1. Ivancevic AM, Kortschak RD, Bertozzi T, Adelson DL. Horizontal transfer of BovB and L1 retrotransposons in eukaryotes. *Genome Biol.* **19**, (2018).

2. Kimura M. A simple method for estimating evolutionary rates of base substitutions through comparative studies of nucleotide sequences. *J. Mol. Evol.* **16**, 111-120 (1980).

3. Li WH. Unbiased estimation of the rates of synonymous and nonsynonymous substitution. *J. Mol. Evol.* **36**, 96-99 (1993).

4. Guéguen L, Duret L. Unbiased Estimate of Synonymous and Nonsynonymous Substitution Rates with Nonstationary Base Composition. *Mol. Biol. Evol.* **35**, 734-742 (2017).

5. Kaehler BD, Yap VB, Huttley GA. Standard Codon Substitution Models Overestimate Purifying Selection for Nonstationary Data. *Genome Biol. Evol.* **9**, 134-149 (2017).

6. Kryazhimskiy S, Plotkin JB. The Population Genetics of dN/dS. *PLoS Genet.* **4**, e1000304 (2008).
