# Supplementary material for: Horizontal transfer and evolution of transposable elements in vertebrates
Source: Nat Commun. 2020 Mar 13;11:1362. doi: 10.1038/s41467-020-15149-4 (PMC7070016; doi:10.1038/s41467-020-15149-4)
Supplement: Supplementary file 4 — Description of Additional Supplementary Files [file 41467_2020_15149_MOESM4_ESM.docx]

**Description of Additional Supplementary Files**

File name: Supplementary Data 1

Description: information on the genome sequences used in the study, including GenBank accession numbers.

File name: Supplementary Data 2

Description: classification and consensus sequences of transposable elements families that were annotated by Repeat Modeler on the genomes used in the study. The “false_positive” column indicates whether a family was excluded because its consensus is partly homologous to a non-TE gene (see main text). A Question mark denotes the failure to classify an element.

File name: Supplementary Data 3

Description: composition in transposable elements of the genomes used in the study.

File name: Supplementary Data 4

Description: hits between transposable elements resulting from horizontal transfer. Each hit involves two TE copies (“copy1” and “copy2”) from two species (“sp1” and “spe2). Copy names combine the following fields separated by colons: contig/scaffold identifier, start and end of the TE copy on the contig/scaffold, and strand. Columns “family1/2” refer to the TE family names generated by Repeat Modeler, whose consensus sequences was used to retrieve the copies from genomes. Columns “pID” to “sEnd” correspond to hit properties reported by blastn. “Ka” and “Ks” columns list mutation rates computed on aligned protein-coding regions, whose length is noted in the “length.aa” column (see Materials and Methods). A “community” or “hitgroup” refers to the clustering procedure (see Material and Methods) and is given an integer number. Column “independent” tells whether the hit belongs to a hit group that was not “explained” by other hit groups (see Material and Methods).

File name: Supplementary Data 5

Description: timetree of the species used in this study, using divergence times from timetree.org. This file is in newick format.
