# Supplementary material for: Horizontal transfer and evolution of transposable elements in vertebrates
Source: Nat Commun. 2020 Mar 13;11:1362. doi: 10.1038/s41467-020-15149-4 (PMC7070016; doi:10.1038/s41467-020-15149-4)
Supplement: Supplementary file 2 — Peer Review [file 41467_2020_15149_MOESM2_ESM.pdf]

Reviewers' comments:

Reviewer #1 (Remarks to the Author):

Zhang et al. provide a very interesting and novel analysis of a broad survey of transposable elements across a collection of 307 vertebrate species, comparing the evolutionary dynamics of elements that are present as a result of horizontal transfer with those from vertical transmission. The major result of the paper is the finding that DNA transposable elements that they examined show purifying selection when the result of horizontal transmission, but not under within species transposition, while retrotransposons generally are under purifying selection independent of the mode of diversification.

I believe that the results of the analysis are generally of interest to researchers in fields of evolutionary biology, genome biology and genome architecture, and the biology of repetitive elements. The results may further strengthen our understanding of the role of transposable elements and their diversification on the regulation of genes and changes in genome structure.

Major comments:

The fundamental basis for the major result of the paper is the dN/dS analysis. Given the wide diversity of sources of sequence information, the diversity of genomic environments, and the large time scales involved in the evolution of those sequences, I have concerns about the reliability of this analysis using a simple pair-wise comparison. There have been concerns raise about the impact of rate stationarity and differences in GC content and their impact on the estimates of the selection parameter (see Huttley 2009 doi:10.1071/ZO09049). Because of the wide range of species involved in this study, I believe this is likely to be a potential issue. It would be good if the authors would be able to control from possible interactions between these confounders and the selection parameter.

It is unclear to me how the authors considered active versus inactive elements in the analysis. From the premise of the work, one would expect that inactive elements (truncated or in the case where cis-preference is an aspect of the elements' biology) that selection would be relaxed or completely lost. It does not appear that the authors address this issue.

In the paragraph from line 436 to 452 the explanation is quite unclear. I would recommend rewriting this using the clade tree topology as a framework for the description, rather than exemplar names. The common terminology of root, subtree, outgroup etc would make this much clearer.

The authors use RepeatModeller and RepeatMasker for the identification of repeats. Neither of these tools have been peer reviewed and both have defects and biases that vary depending on the version being used. In the case of RepeatModeller artifactual consensus sequences have been seen at high frequency and with RepeatMasker there is an underlying implementation that focuses on human repeats at the expense of more general repeat identification. It would be worthwhile for the authors to indicate how carefully the resulting elements identified in the survey were curated to control for these defects in the tooling.

The code provided in the scripts zip file would benefit from being cleaned up so that lines are formatted with the view to being read on conventionally wide text editors; currently code lines in the zip commonly exceed 200 columns (c.f. the conventional <100 columns). These deficits in conjunction with the coding style and lack of clear documentation on many functions make reviewing the code extremely difficult.
The code should also have absolute paths within the researchers' file systems changed to relative or relocatable paths so that the code can be run on other people without onerous levels of code editing.

Minor comments:

On line 47, there is a claim that TEs "make up most of" the chromosomes. This is an overstatement; they are certainly a significant component, but by no means most in most species.

On line 122, the word "essentially" does not add anything to the meaning of the sentence, and in my view detracts.

On line 392, it is unclear what is meant by "we confronted every two communities". This should be rewritten to be more clear.

The version of all software tools used should be recorded in the manuscript; the link in the bibliography to RepeatMasker and RepeatModeller are to 4.0 and 1.0 respectively. The patch-level version number is not included, but should be.

Reviewer #2 (Remarks to the Author):

This manuscript represents an impressive piece of work by the authors, who have expanded their previous analyses of insect HTT to vertebrates and improve the methods along the way.

I like the work and think that it should eventually be published but I have several criticisms that must be addressed.

All of the major questions that I have involve the classification of the TEs evaluated. First, I am frustrated by the fact that the authors trusted the raw repeatmodeler output as their method for classifying TEs. While repeatmodeler is a nice software package and serves as an excellent start to the process, it makes numerous mistakes in it's classifiction efforts. My own experience has demonstrated that a significant number of TEs are misclassified by repeatmodeler and this could lead to errors as the authors report general trends in the types of TEs that are the subject of HTT events. I doubt that it will have a major impact, especially given that the observations they make are in line with previous studies. However, it would be nice to see some work given to determining how accurate the classifications given by repeatmodeler actually are. Even just taking a subset of repeatmodeler output and subjecting it to manual classifications would provide an estimate of this value and inform the validity of overall conclusions.

In a further, related criticism, repeatmodeler is known to sometimes fragment TEs. For example, a long 600 bp L1 may be detected by repeatmodeler as two or three separate fragments rather than just a single model. As such, it's possible that, if that L1 was the subject of HTT, it could have been counted as many as three times. Were these sorts of events taken into account? If so, how?

Along similar lines, I also have concerns about the accuracy of the results based on the data2-TE_families.txt file. I examined it and notices that many of the models were completely unclassified. For example, I count 92503 entries with "?" in place of classifications (Class I/Class II, DNA, LTR, etc.). It appears most of these came from Dfam rather than Repbase. The problem is that such entries represent nearly 37% of the TEs evaluated. So, over 1/3 of the TEs were not identified as belonging to a particular class. It's unclear whether there is a pattern to that lack of classification. Were DNA transposons more likely to be unclassified? LTR retrotransposons? If so, then calculations based on those counts could be erroneous. The authors need to acknowledge this potential bias and address how it was accounted for.

Other than these major criticisms, I very much like the manuscript and want to see it published as soon as is feasible. Minor editorial comments are below:

Line 82 - change to "allowed us"
Line 105 - change to "TE copies"
Line 147 - change to "brought into their hosts"
Line 224 - change to "the fact that Tc1/Mariner"
Line 279 - change to "LTR retrotransposon"
Line 356 - change to "Ks distribution"
Line 352-354 - the sentence beginning with, "To reduce the workload,..." is very confusing. I think I understand what is being said. If so, I think the confusion could be reduced by changing "that with" in line 354 to "the one with".

Reviewer #3 (Remarks to the Author):

In this manuscript the authors report the identification and characterisation of hundreds of candidate HTT events based on a sophisticated analysis pipeline that at its core is based on finding more sequence homology from TEs across species than within species.

There are 4 major results reported: 1, the vast majority of HTT are among ray-finned fishes, 2, HTT incurs purifying selection on all TE types, 3, DNA transposons evolve neutrally within genomes in the absence of HTT, unlike most retrotransposons which evolve under purifying selection, and 4, host taxa have a strong impact on HTT frequency in animals.

These results are both novel and of interest to a wide cross section of biologists.

The methods used for identification of candidate HTT events appear to be sound, however it is difficult to know how reproducible they might be in the hands of other researchers given their complexity. It is not clear from the methods described if all code used for the analysis is publicly available.

I have some reservations about the methods used to determine purifying selection. Based on my reading of the manuscript, the authors use sequence comparisons of TE pairs to calculate Ka and Ks. This is approach is fine when comparing proteins/loci between species, but is of questionable validity when comparing proteins/loci within populations/species (see Kryazhimskiy S, Plotkin JB (2008) The Population Genetics of dN/dS. PLoS Genet 4(12): e1000304. doi:10.1371/journal.pgen.1000304). The problem I have with this manuscript is that is not clear to me if all the Ka Ks rates are derived from comparison of TE pairs between species or if some are derived from comparisons within the TE population of a single genome. It is not clear to me based on my reading of the methods if all comparisons are between species. If Ka Ks calculations are not all from between species/genome comparisons than the conclusions drawn regarding purifying selection vs neutral substitution rates may be invalid.

I have one additional reservation based on figure 1, which summarises the HTT events identified in the manuscript. This figure shows virtually no HTT events from retrotransposons in mammals/vertebrates, however this is in stark contrast to work we have previously published looking specifically at BovB and L1 HTT (Ivancevic et al, cited in manuscript). It would be useful to have some discussion of this discrepancy, particularly if a likely methodological explanation can be proferred for it.

I have one final comment regarding the habitat swapping permutation test to compare the frequency of HTT events between fishes and aquatic tetrapods. The authors conclude that there is little evidence to suggest that aquatic habitat is more conducive to HTT and that the excess of events observed in ray-finned fishes is host genome dependent. However they acknowledge that the power of their test is limited by the small number of aquatic tetrapod species in their dataset. Because of this limitation, I think the jury is still out on the hypothesis that an aquatic environment is more conducive to HTT than

a terrestrial environment. I suggest that the authors be more conservative in their interpretation of these results.

I have a few minor corrections to suggest:

line 82: "allowed to establish" delete "allowed to" and change to "established"

line 105: "TE copies" change to "TE copy"

line 122: change "considered to essentially result" to "considered to result"

line 285: change "indicating" to "suggesting"

This review written by David Adelson of the University of Adelaide.

Reviewers' comments:

Reviewer #1 (Remarks to the Author):

Zhang et al. provide a very interesting and novel analysis of a broad survey of transposable elements across a collection of 307 vertebrate species, comparing the evolutionary dynamics of elements that are present as a result of horizontal transfer with those from vertical transmission. The major result of the paper is the finding that DNA transposable elements that they examined show purifying selection when the result of horizontal transmission, but not under within species transposition, while retrotransposons generally are under purifying selection independent of the mode of diversification.

I believe that the results of the analysis are generally of interest to researchers in fields of evolutionary biology, genome biology and genome architecture, and the biology of repetitive elements. The results may further strengthen our understanding of the role of transposable elements and their diversification on the regulation of genes and changes in genome structure.Major comments:

The fundamental basis for the major result of the paper is the dN/dS analysis. Given the wide diversity of sources of sequence information, the diversity of genomic environments, and the large time scales involved in the evolution of those sequences, I have concerns about the reliability of this analysis using a simple pair-wise comparison.

We understand these concerns. However, the data forced us to base our Ka Ks analysis on pair-wise comparisons because we practically cannot build TE trees on which we could measure Ka and Ks rates. Due to the high degradation (and very high number) of the TEs, it is already quite difficult to make trees. And to automatically make trees for which we could measure Ka and Ks ratio would be even harder, given the issues related to frameshifts.
The couple of studies that analyzed selection acting on TEs manually edited single alignments of conserved TEs. Our work is the first to analyze selection on TEs as such a scale. We now clearlystate the challenges we had to cope with and the limitations of our procedure (line 255) "*The degradation of TE protein-coding regions by random mutations (many of which cause frameshifts) and the sheer number of TE copies imposed great challenges to this analysis. Our automatic procedure (detailed in the Methods section) was designed to overcome these challenges at the expense of measuring Ka and Ks mutation rates on pair-wise, rather than on multiple, sequence alignments.*"

With respect to the timescale involved, we do not view them as very large since we selected hits between TEs that show relatively high sequence identity (>75%) and moderate Ks (< 0.5).

There have been concerns raise about the impact of rate stationarity and differences in GC content and their impact on the estimates of the selection parameter (see Huttley 2009 doi:10.1071/ZO09049). Because of the wide range of species involved in this study, I believe this is likely to be a potential issue. It would be good if the authors would be able to control from possible interactions between these confounders and the selection parameter.

Our inferences are supported by comparisons between Ka/Ks ratios, not just by absolute values of selection parameters. These comparisons do control for confounding factors, and are (1) lower Ka/Ks ratios between TEs that diverged through HTT compared to those that diverged within genomes, and (2) Ka/Ks ratios significantly < 1 for most retrotransposons that diverged within-genome, while it is not the case for DNA transposons. The two presumed factors explaining these differences are the mechanism of TE divergence (HTT vs transposition only) and TE biology (retrotransposons vs DNA transposons), both incurring difference in selective pressure. We do not see any credible confounding factor that could contribute to (1) or (2).
In respect to (1), we used the same TEs copies (hence host species) to measure Ka/Ks during divergence via HTT and during divergence within genomes. In both cases, these TEs are those composing the 775,801 retained hits (although these TE and species obviously do not form the *pairs* when it comes to HTT vs. within genome divergence). Therefore, host genomes and TEs are not confounding factors. The only confounding factor we can think of is the following: TEs that diverged through HTT belong to different species as opposed to those that diverged within genome. These species may differ in their usage of synonymous codons, and this difference (rather than selection on functional TEs during HTT) could somehow reduce Ka/Ks ratios.

We therefore assessed how Ka/Ks ratios depended on the difference in GC contents between species involved in HTT, which we used as a proxy for differences in synonymous codon usage or other effects related to genomic composition. We have made the plots below for the three TE subclasses:

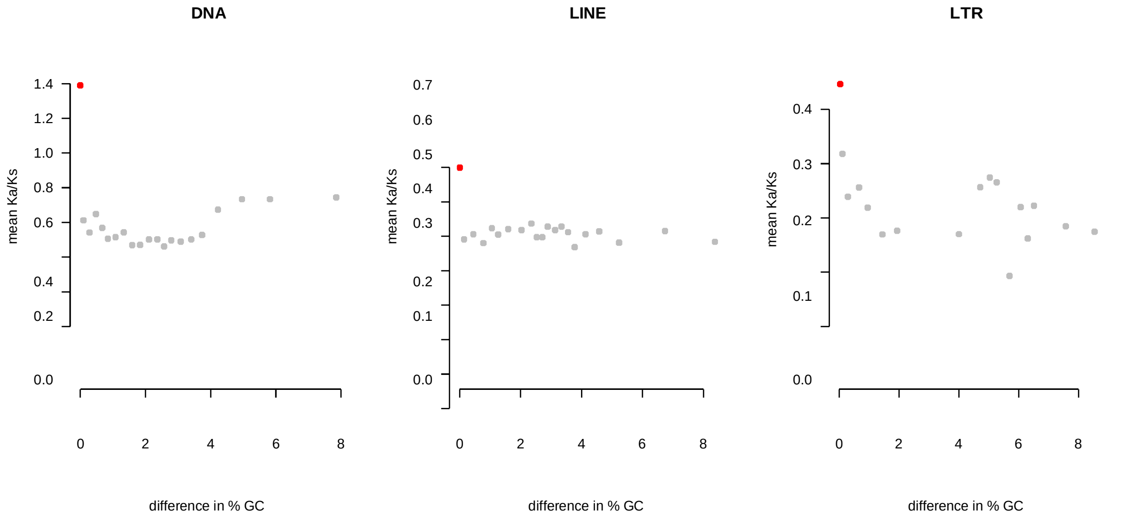

In each plot, a grey point is the mean Ka/Ks ratio (weighted by alignment lengths) across TEs transferred between species whose differences in %GC content belong to a particular class (we made 20 classes per plot, for readability). Points in red correspond to mean Ka/Ks ratio for TEs that diverged within genomes (hence no difference in % GC). We can clearly see that the difference in % GC (and presumably in codon usage) has much lower impact on Ka/Ks ratios than HTT vs non-HTT. Since this potential confounding factor seems far-fetched and has no visible impact, we would rather not mention it in the paper.

As for (2), Ka/Ks estimates on DNA transposons and retrotransposons used the same species overall (essentially ray-finned fishes, which contributed most of the TE copies used in the computations), so we do not see what confounding factor could explain the differences (the one mentioned above does not apply).

We also tried to investigate the influence of GC content on Ka/Ks ratios (we could not come up with a way to estimate and control rate non-stationarity). We took the ~4.6M Ka/Ks ratios between TEs that diverged within the same genome (it seems difficult to test for the influence of GC content on the inferred mutation rates of TEs that moved between species). We averaged Ka/Ks ratios per genome and TE superfamilies and tested for a correlation between these means and GC contents. We found no significant correlation (r = 0.07630066, t = 1.8946, df = 613, p-value = 0.05861) across all superfamilies and no significant correlation within superfamilies ($p > 5\%$). The correlation is even lower when it uses average Ka/Ks ratios per hit group and species (r = 0.000241742, t = 1.2882, df = 6863, p-value = 0.1977).

It is unclear to me how the authors considered active versus inactive elements in the analysis. From the premise of the work, one would expect that inactive elements (truncated or in the case where cis-preference is an aspect of the elements' biology) that selection would be relaxed or completely lost. It does not appear that the authors address this issue.

We think that we considered this issue, as we state at lines 363 "*This procedure effectively reconstructs (partial) homologous protein-coding sequences from a pair of TEs and investigates traces of selection that acted on these sequences before their deactivation*". This indicates that we did not consider active versus inactive elements in the analysis. To make this point more salient, we have moved this sentence to the result section (line 259), which required some rewording: "*These mutation rates can reveal traces of selection that acted on TE protein-coding sequences before the potential deactivation of their encoded product*".
Lines 271+ also refers to the effect raised by the reviewer: "*These higher Ka/Ks ratios do not reflect a failure to detect traces of selection on pairs of TEs that have been non-functional for a longer time* […]. *Actually, the investigated pairs of TEs that diverged by transposition within genomes show lower Ks values than TEs that diverged by HT (Table S2)*". We think that this argument makes clear that the effect of TE deactivation can only reduce the power to detect purifying selection. Our ability to reveal selective patterns despite the potential action of non-selected mutations masking traces of earlier selection indicates that we do not need to consider active TEs specifically. To clarify this point, we

slightly expanded on this (line 273): "*Indeed, the investigated pairs of TEs that diverged by*

transposition within genomes show lower Ks values than TEs, hence diverged more recently, than TEs that diverged by HT (Table S2) ".
We however agree that relaxed selection after TE deactivation can make difficult the detection of purifying selection, especially if selection was weak. This might have been the case for Jockey elements, which are the only retrotransposons that show no evidence for purifying selection during transposition (Ka/Ks not significantly < 1 on average). Hence, we have checked whether Ka/Ks tended to be lower between less divergent copies of Jockey TEs (which would have shorter divergence time after their potential deactivation). We found no such trend (see the plot below, where each point represents a hit group of Jockey TEs that diverged within genomes).

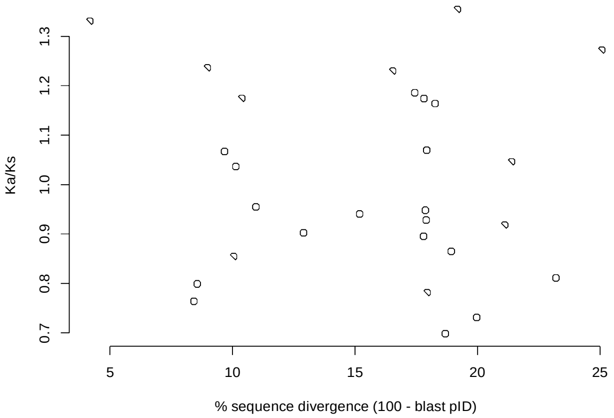

Therefore, we decided not to do specific analyses on active TEs, which we are not able to reliably pinpoint anyway since a protein's activity cannot be ascertained from its mere sequence. We would rather not report the figure shown above, as we believe it would distract readers form our main results and be detrimental to the paper.

In the paragraph from line 436 to 452 the explanation is quite unclear. I would recommend rewriting this using the clade tree topology as a framework for the description, rather than exemplar names. The common terminology of root, subtree, outgroup etc would make this much clearer.

We agree that the paragraph is difficult to understand, but this reflects the complexity of the procedure described, hence the use of a supplementary figure dedicated to this procedure. We carefully considered the reviewer's comment, but decided not to implement the recommendations for the following reasons.

- The procedure is illustrated by supplementary figure 4, which uses exemplar names for the clades. We believe that exemplar names are required to avoid ambiguities as to which entities the text refers to. We tried and failed to explain this complex procedure without giving exemplar names. Following the reviewer's recommendation results in a rather difficult explanation where we would have to refer to "a subtree", "another subtree", a "third subtree", etc.
- As we only refer to monophyletic subtrees, we think the word "clade" (a very common term in phylogenetics that we use throughout) is more appropriate.
- As for "root" and "outgroups", we do not think that these words would help, as our procedure does not rely on these entities.

That said, we remain open to propositions to improve the clarity of this section.

The authors use RepeatModeller and RepeatMasker for the identification of repeats. Neither of these tools have been peer reviewed and both have defects and biases that vary depending on the version being used. In the case of RepeatModeller artifactual consensus sequences have been seen at high frequency and with RepeatMasker there is an underlying implementation that focuses on human repeats at the expense of more general repeat identification. It would be worthwhile for the authors to indicate how carefully the resulting elements identified in the survey were curated to control for these defects in the tooling.

In respect to artifactual consensuses, by which we suppose the reviewer refers to consensus sequences that do not correspond to TEs, we think the method description makes clear that basically none could have ended up in the TEs we selected. Namely:

- We selected elements that were classified down to the super-family level (line 337).
- We searched for TE consensus sequences (TE families) that showed homology to non-TE proteins and discarded them (line 334).
- to detect HTT, we only retained hits between TEs assigned to the same superfamily, which constituted ~98% of the hits. In response to reviewer 2's comment (see below), we now highlight this result to argue for the accuracy of the automatic TE classification (line 346).
- From these hits, we computed Ka and Ks ratio between TEs (within and between species) to retain alignments longer than 100 codons (300 bp) (lines 386-389). The fact that these ratios are generally < 1 for HTT shows that we did not align random sequences.

It seems difficult for a spurious TE to pass all these filters. More generally, HT between animals has only been documented for TEs, so a non-TE element somehow classified as a TE would be very unlikely have an influence on our results.

Regarding annotation biases, RepeatMasker used consensuses generated by RepeatModeler for each species to locate TE copies, so we do see how it might have favored human-type repeats in our analysis.
We now make clearer that all the statistics we report pertain to the TEs that we were able to automatically classify, not necessarily all the TEs in the studied species (line 154) "*Our automatic annotation procedure may also have missed TEs present in genomes, thus we stress that the numbers we report only apply to elements that were successfully classified into superfamilies*". We still want to emphasize that our automatic procedure, despite its potential shortcomings, was specifically designed to reduce TE annotation biases. It is not realistic for a single study to "manually" annotate TEs in so many genomes, and the use of already annotated TEs would have induced a huge bias favoring model species. Sequence annotation always comes with bias, as it relies on previous annotation efforts that greatly vary across taxa and sequence types.

The code provided in the scripts zip file would benefit from being cleaned up so that lines are formatted with the view to being read on conventionally wide text editors; currently code lines in the zip commonly exceed 200 columns (c.f. the conventional <100 columns). These deficits in conjunction with the coding style and lack of clear documentation on many functions make reviewing the code extremely difficult.
The code should also have absolute paths within the researchers' file systems changed to relative or relocatable paths so that the code can be run on other people without onerous levels of code editing.

We have restructured the pipeline into several script files that more clearly indicate the stages of the analysis and we have improved the code documentation by adding many explanatory comments. We have also removed many (uncommented) functions that were actually not used in the analysis, and changed file paths to relative paths.
As for line length, many text editors soft-wrap lines automatically, so does RStudio, a free cross-platform application that we view as the tool of choice to review and edit R code. So, we do not see line length as an issue that needs to be addressed, considering the time required compared to the uncertain benefits of line shortening.
We understand that the code we provide may not follow best practices, but since this is not a methodological paper (and because none of its authors is a software developer), the provided code is not intended as a ready-to-use piece of software. The method description is what should be used to reproduce the study. As for the scripts, they are provided to help researchers implement their own analyses based on ours, after some code editing.
We would also like to stress that we provided the code for the almost complete pipeline (only few steps were not scripted), down to the generation of the manuscript's figures and tables, which inevitably lengthens code review. Similar studies usually only provide scripts for specific parts of the analysis.

Minor comments:

On line 47, there is a claim that TEs "make up most of" the chromosomes. This is an overstatement; they are certainly a significant component, but by no means most in most species.

We have rephrased as follows "*This property has allowed TEs to invade the genomes of virtually all organisms, of which they can make up most of the chromosomes*" (line 47).

On line 122, the word "essentially" does not add anything to the meaning of the sentence, and in my view detracts.

We removed the word.

On line 392, it is unclear what is meant by "we confronted every two communities". This should be rewritten to be more clear.

We rewrote as follows "*In the second iteration, we generated pairs of communities to evaluate if hits composing the two communities of a pair could result from a single HTT event* " (line 413).

The version of all software tools used should be recorded in the manuscript

We followed this recommendation.

the link in the bibliography to RepeatMasker and RepeatModeller are to 4.0 and 1.0 respectively. The patch-level version number is not included, but should be.

We followed this recommendation.

Reviewer #2 (Remarks to the Author):

This manuscript represents an impressive piece of work by the authors, who have expanded their previous analyses of insect HTT to vertebrates and improve the methods along the way.

I like the work and think that it should eventually be published but I have several criticisms that must be addressed.

All of the major questions that I have involve the classification of the TEs evaluated. First, I am frustrated by the fact that the authors trusted the raw repeatmodeler output as their method for classifying TEs. While repeatmodeler is a nice software package and serves as an excellent start to the process, it makes numerous mistakes in it's classifiction efforts. My own experience has demonstrated that a significant number of TEs are misclassified by repeatmodeler and this could lead to errors as the authors report general trends in the types of TEs that are the subject of HTT events. I doubt that it will have a major impact, especially given that the observations they make are in line with previous studies. However, it would be nice to see some work given to determining how accurate the classifications given by repeatmodeler actually are. Even just taking a subset of repeatmodeler output and subjecting it to manual classifications would provide an estimate of this value and inform the validity of overall conclusions.

We now argue for the reliability of the repeatModeler classification by indicating the very high (~98%) proportion of hits involving TEs of the same superfamily (line 346). Since TEs from different genomes were classified independently in separate repeatModeler runs, we believe that this high proportion indicates that the vast majority of TEs were correctly classified.
In the same vein, we now indicate that >99% of TE copies had a hit to a protein that belonged to the family inferred by repeatModeler (line 353).

In a further, related criticism, repeatmodeler is known to sometimes fragment TEs. For example, a long 600 bp L1 may be detected by repeatmodeler as two or three separate fragments rather than just a single model. As such, it's possible that, if that L1 was the subject of HTT, it could have been

counted as many as three times. Were these sorts of events taken into account? If so, how?

Although it is not explicit in the manuscript, TE fragmentation is taken into account by our hit clustering procedure, since it is designed to work on incomplete TE copies. Fragments of the same TE (seen as distinct copies) would generate hits to a unique TE copy, or to closely related TE copies, from other genomes. These hits would therefore end up in the same hit group (hence same HTT event), due to the similarity between the other genomes' copies (our criterion 1 of hit clustering would be passed). TE fragmentation can only be an issue if it occurred similarly in all genomes that share the TE. This is highly unlikely to happen for a TE that underwent HTT. Even under this scenario, hits between TE fragments should still pass criterion 1 (and end up in the same hit group), thanks to our procedure that accounts for the possibility that non-homologous TE copies be non-overlapping parts of a single TE that underwent differential degradation (groups of hits between TE copies that show no similarity at the DNA or protein level pass criterion 1). We now make this point clear at line 418 : "*As in (25), if the sets of TE copies composing the two hit communities did not show any DNA sequence homology (no blast hit) and insufficient (<100 bp) protein sequence identity, we considered that these sets of copies could represent non-overlapping parts of a TE that underwent fragmentation or differential degradation within genomes after a unique transfer*".

Along similar lines, I also have concerns about the accuracy of the results based on the data2-TE_families.txt file. I examined it and notices that many of the models were completely unclassified. For example, I count 92503 entries with "?" in place of classifications (Class I/Class II, DNA, LTR, etc.). It appears most of these came from Dfam rather than Repbase. The problem is that such entries represent nearly 37% of the TEs evaluated. So, over 1/3 of the TEs were not identified as belonging to a particular class. It's unclear whether there is a pattern to that lack of classification. Were DNA transposons more likely to be unclassified? LTR retrotransposons? If so, then calculations based on those counts could be erroneous. The authors need to acknowledge this potential bias and address how it was accounted for.

As now indicated in the manuscript (line 155) "*we stress that the numbers we report only apply to elements that were successfully classified into superfamilies*". In that sense, calculations are not erroneous, they are just restricted to TEs that could be annotated. We do not base our conclusions regarding the relative contributions of different TE types to HTT on the raw numbers of HTT events. Indeed, we contrast the numbers of HTT events with the numbers of annotated TE copies, both being shown side-by-side on figure 4. For instance, we say that "*Besides Tc1/Mariner, the non-LTR retrotransposon Rex-Babar stands out as being the superfamily involved in the second highest number of transfers, with one of the highest ratios of transfers per copy*".
More generally, the high proportion of unclassified repeats is a common result of de novo TE annotation procedures. For example, Petersen al. 2019 (https://doi.org/10.1186/s12862-018-1324-9) performed TE annotation in 62 insect genomes and found that >50% of the TE consensuses were not classified in most species. Unclassified consensuses may correspond to ancient highly degraded TEs or to non-TE repeats. Classifying these elements is a difficult task associated with some degree of uncertainty (De Koning et al. 2011, https://doi.org/10.1371/journal.pgen.1002384), and which requires developing specific procedures that are beyond the scope of this study.
We would also like to point out that this study uses a much greater number of TEs per species than previous studies on HTT (which focused on specific TE types, full-length elements or active elements, all constituting a very small portion of the TE repertoire).

Other than these major criticisms, I very much like the manuscript and want to see it published as soon as is feasible. Minor editorial comments are below:
Line 82 - change to "allowed us"

We followed this recommendation.

Line 105 - change to "TE copies"

We assumed that the reviewer meant "TE copy" (as suggested by Dr. Adelson below) and applied the change.

Line 147 - change to "brought into their hosts"

Line 224 - change to "the fact that Tc1/Mariner"
Line 279 - change to "LTR retrotransposon"
Line 356 - change to "Ks distribution"

We followed these recommendations.

Line 352-354 - the sentence beginning with, "To reduce the workload,..." is very confusing. I think I understand what is being said. If so, I think the confusion could be reduced by changing "that with" in line 354 to "the one with".

We followed this recommendation and slightly reworded the sentence for clarity "*To reduce the workload, Ks distributions involving two sister clades older than 250 My used just one genome per subclade younger than 30 My, the one with the highest number of annotated core genes*".

Reviewer #3 (Remarks to the Author):

In this manuscript the authors report the identification and characterisation of hundreds of candidate HTT events based on a sophisticated analysis pipeline that at its core is based on finding more sequence homology from TEs across species than within species.

There are 4 major results reported: 1, the vast majority of HTT are among ray-finned fishes, 2, HTT incurs purifying selection on all TE types, 3, DNA transposons evolve neutrally within genomes in the absence of HTT, unlike most retrotransposons which evolve under purifying selection, and 4, host taxa have a strong impact on HTT frequency in animals.

These results are both novel and of interest to a wide cross section of biologists.

The methods used for identification of candidate HTT events appear to be sound, however it is difficult to know how reproducible they might be in the hands of other researchers given their complexity. It is not clear from the methods described if all code used for the analysis is publicly available.

We have deposited all the code into GitHub (https://github.com/jeanlain/HTvertebrates) and now make clear that all the code used is publicly available (line 519).

I have some reservations about the methods used to determine purifying selection. Based on my reading of the manuscript, the authors use sequence comparisons of TE pairs to calculate Ka and Ks. This is approach is fine when comparing proteins/loci between species, but is of questionable validity when comparing proteins/loci within populations/species (see Kryazhimskiy S, Plotkin JB (2008) The Population Genetics of dN/dS. PLoS Genet 4(12): e1000304. doi:10.1371/journal.pgen.1000304). The problem I have with this manuscript is that is not clear to me if all the Ka Ks rates are derived from comparison of TE pairs between species or if some are derived from comparisons within the TE population of a single genome. It is not clear to me based on my reading of the methods if all comparisons are between species. If Ka Ks calculations are not all from between species/genome comparisons than the conclusions drawn regarding purifying selection vs neutral substitution rates may be invalid.

Interpretations from intraspecific Ka Ks rates are indeed problematic if these rates are measured between alleles. However, none of the Ka-Ks rates computed in this study were measured between alleles. We measured them between different copies, hence different loci (paralogs). Almost all the mutations that differentiate TE copies should be fixed in the species and are not polymorphic. Ka and Ks rates measured between paralogs are commonly interpreted in terms of selective pressures. Therefore, we do not think we need to modify our text in response to this comment.

I have one additional reservation based on figure 1, which summarises the HTT events identified in the manuscript. This figure shows virtually no HTT events from retrotransposons in mammals/vertebrates, however this is in stark contrast to work we have previously published looking specifically at BovB and L1 HTT (Ivancevic et al, cited in manuscript). It would be useful to have some discussion of this discrepancy, particularly if a likely methodological explanation can be proffered for it.

By looking at figure 1 in Ivancevic et al, we could locate 4 BovB transfers between mammals and other vertebrates (squamates). We looked into our data and found 10 hit groups of BovB elements linking mammals and squamates and which comprised enough TE copies to exclude contamination. However, just one of them (visible on Figure 2) passed the filter described at line 445. See below the histograms of the Ks of the 0.5% of core genes with lowest Ks, and of TEs for one of the non-retained hit groups.

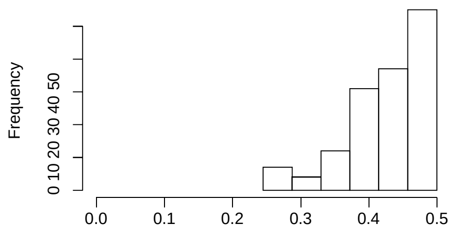

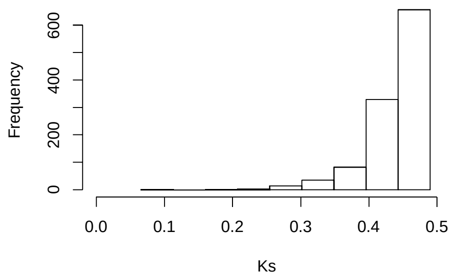

From these graphs, it is difficult to exclude the hypothesis that the Ks values of TEs and core genes are left tails of similar distributions (we cannot statistically test this because Ks values are not independent, they are not branch-based). The retained TE hits, whose Ks values are lower than 99.5% of core gene Ks, may just represent the most conserved vertically-inherited TEs. We agree that the patchy taxonomic distribution of these TEs limited to squamates, ruminants and marsupials is suggestive of HTT. However, the assessment of patchiness is complex and somewhat subjective as we do not know the probabilities of losses of TEs. Hence, we do not use this criterion in our large-scale analysis.

We would rather not discuss the BovB case in the paper, as readers might not understand why we would focus on a particular HTT event. We think that the statement "*our extremely stringent filters likely discarded many hits that did not actually result from vertical TE inheritance*" (line 152) is sufficient.

We did not find any L1 HTT between mammals and other vertebrates on figure 1 in Ivancevic et al, which is consistent with the absence of such HTT in our study.

I have one final comment regarding the habitat swapping permutation test to compare the frequency of HTT events between fishes and aquatic tetrapods. The authors conclude that there is little evidence to suggest that aquatic habitat is more conducive to HTT and that the excess of events observed in ray-finned fishes is host genome dependent. However they acknowledge that the power of their test is limited by the small number of aquatic tetrapod species in their dataset. Because of this limitation, I think the jury is still out on the hypothesis that an aquatic environment is more conducive to HTT than a terrestrial environment. I suggest that the authors be more conservative in their interpretation of these results.

We agree with this comment. We did not mean to favor a particular hypothesis regarding the excess of HTT in ray-finned fishes (we do not think our text does). To make this clearer, we have added the following (line 214) "*A more powerful test of the hypothesis that aquatic environments favor HTT would require aquatic and non-aquatic species of multiple evolutionary origins.*"

I have a few minor corrections to suggest:

line 82: "allowed to establish" delete "allowed to" and change to "established"

We have decided to follow reviewer 2's recommendation regarding this sentence.

line 105: "TE copies" change to "TE copy"

line 122: change "considered to essentially result" to "considered to result"

We have implemented these recommendations.

line 285: change "indicating" to "suggesting"

We felt that this change yielded an inelegant repetition of “suggesting”, so we tempered the phrase as follows: "*which may indicate lower cis-preference of these elements compared to other retrotransposons*" (line 299).

This review written by David Adelson of the University of Adelaide.

Reviewers' comments:

Reviewer #1 (Remarks to the Author):

I am not convinced the authors have made sufficient improvements/changes to the manuscript to address the concerns that I raised in my original review.

Addressing the points in order:

1. Regarding the issues around dN/dS analysis as a simple distance metric, the additional text does explain why the approach was chosen, but does not adequately detail the potential impacts that this choice of approach has on the reliability of the analysis. In the second response paragraph, the authors talk about using Ka/Ks ration rather than absolute values. This appears to miss the point of the findings reported by Huttley which looks at the impacts of non-stationarity on this very ratio. Further, I would argue that 75% identity for a genetic element under some form of selection is actually quite distant. However, the figures presented in the rebuttal do go towards satisfying the concern. I would hope that a brief comment on the issue be included in the manuscript and the figures be included in supplemental material.

2. The authors have missed the point of my concern regarding inactive/degraded elements. I think the impact should be considered, but as the authors state in their letter, "This indicates that we did not consider active versus inactive elements in the analysis." I feel that making the claims that they do about the partial reconstruction would require having a model of the evolutionary topology of the elements, which the authors do not have. Preferring not to comment on this issue in the paper is odd.

3. If the authors do not wish to revise the text for the description of "Hit group evaluation", I would strongly recommend providing in the supplemental material a pseudocode representation of the process, to go with a diagramatic representation of the HTT system being assessed in supp figure 4.

4. Accepted.

5. I remain unable to have time to review the code because of the lack of clear structure and formatting. While the manuscript is not a methodological paper, in computational analysis, the devil is in the details. It is very troubling that the analysis is not presented in a form that would be understood to allow perfect replication of the analysis and for others to clearly read the approach.

Reviewer #2 (Remarks to the Author):

The authors have done an excellent job addressing my concerns. I appreciate their diligence. I have no additional comments and recommend acceptance of the manuscript.

Reviewer #3 (Remarks to the Author):

The authors have responded constructively to my comments, but I still have a concern regarding the Ka/Ks analysis within genomes. My original concern was that Ka/Ks measurements for TEs within a genome are not appropriate as per the Kryazhimskiy & Plotkin paper doi:10.1371/journal.pgen.1000304) . The authors have responded that this is not an issue because the comparisons made are not between alleles, but this misses the point. The Kryazhimskiy paper specifically talks about comparisons within bacterial species populations as being inappropriate and bacteria are prokaryotic, hence a between allele comparison is impossible. My concern is that individual TEs in a genome are like individual bacterial clones in a population in that they arise at various times in a stochastic fashion. I have taken the liberty of reproducing a relevant paragraph

from the Kryazhimskiy paper below:

"The dN/dS ratio was originally developed for the analysis of genetic sequences from divergent species [1],[4],[5], the differences amoung which represent fixation events along independent lineages. Theoretical work on the relationship between dN/dS and selection likewise assumes that sequences are sampled from independent, divergent species [3], as do computer packages used to estimate dN/dS from data [6],[7]. Nonetheless, the dN/dS ratio test is frequently applied to data that may represent samples from a single population, particularly in the case of microbes (e.g. [3], [8]–[18]). In such cases, the differences between sequences do not represent fixation events along independent lineages, but rather polymorphisms segregating in a single population."

There are additional points made in the paper that raise concerns about using dN/dS within populations, but I am not going to copy and paste the whole thing into this review. The situation described in this paragraph would seem to be equivalent to the situation of individual TEs within a genome. In this case, the Ka/Ks ratio is going to be insensitive to selection and should not be relied upon. I do not think it is appropriate to rely on this method of measuring selection for TEs within a genome/species. Therefore I recommend that the results based on this approach be removed from the paper.

I have one additional comment with respect to the author's response to my query about BovB HTT between mammals and other taxa. Our original method (Ivancevic et al) for detecting BovB HTT was quite stringent and used patchy phylogenetic distribution as one of the criteria because this is a hallmark of HTT. Ascertainment of a patchy distribution requires that no trace of the TE in question can be detected in this genome. While it is true that one cannot estimate the probability of complete removal of all traces of a TE, observations of persistent TE fragments from "fossil" TEs are extremely commonplace so it is reasonable to conclude that complete removal of a particular TE family/subfamily from a genome has an extremely small probability. If the large discrepancy between our results for BovB and the authors' results for BovB primarily differ due to the use of a patchy distribution, then this is something that should be discussed as it represents a profound difference in methodology, particularly since the highly patchy distribution of BovB makes them a useful validation set for other methods. We reported in excess of 50 high confidence HTT events for BovB involving mammalia in our supplementary material so the real question is why are these undetected? Exploration of these discrepancies should result in the authors being able to make their method more sensitive. I stand by my earlier recommendation that this still needs to be discussed.

This reply submitted by David Adelson of the University of Adelaide

Here are our point-by-point responses (in blue) to the reviewer comments. Some articles supporting our claims are listed at the end of the document.
Line numbers refer to the manuscript without track changes.

Reviewer #1 (Remarks to the Author):

I am not convinced the authors have made sufficient improvements/changes to the manuscript to address the concerns that I raised in my original review.

Addressing the points in order:

1. Regarding the issues around dN/dS analysis as a simple distance metric, the additional text does explain why the approach was chosen, but does not adequately detail the potential impacts that this choice of approach has on the reliability of the analysis.

In a new supplementary section titled "*Impact of the pairwise approach on the reliability of the Ka/Ks analysis* ", we detail how our pairwise alignment approach could have affected the analysis. We identified and investigated three possible issues:

- The unsophisticated molecular evolution model we were constrained to might underestimate mutation rates. Under negative selection, this is more likely to affect the Ks than the Ka, hence Ka/Ks ratios may be overestimated between TEs that diverged under negative selection for a long time.
- In the same vein, our model does not account for mutations accumulating after TE deactivation, all of which are necessarily neutral. These mutations would shift Ka/Ks ratios towards 1.
- Non-stationarity in G+C composition cannot be taken into account, which may bias estimates of Ka/Ks.

We made plots that are similar to the ones we sent in our previous response (supplementary figures 3-6). Although we do detect some degree of saturation in the Ks, these plots show that the aforementioned factors cannot explain the broad patterns of variation in Ka/Ks we observe, i.e., the fact that Ka/Ks ratios are <1 for most TEs, that they are lower between TEs involved in HTT and vary depending on TE type.
We nevertheless warn readers against using the Ka and Ks values we report in our supplementary datasets for analyses requiring highly accurate estimates.

In the second response paragraph, the authors talk about using Ka/Ks ration rather than absolute values. This appears to miss the point of the findings reported by Huttley which looks at the impacts of non-stationarity on this very ratio.

We certainly agree that non-stationarity impacts the Ka/Ks ratios. We meant that our conclusions were supported by differences in the Ka/Ks ratios between TE types and modes of TE divergence, not just by the absolute values of Ka/Ks ratios.
We now refer to Huttley's findings (line 273). We did not however find the article suggested by the reviewer (Huttley 2009 doi:10.1071/ZO09049) as the most relevant, since this study focuses on the deep phylogenetic relationships in mammals, rather than on the effects of non-stationarity in base composition on Ka/Ks. We instead quoted Kaehler, Yap and Huttley (2017).

Further, I would argue that 75% identity for a genetic element under some form of selection is actually quite distant. However, the figures presented in the rebuttal do go towards satisfying the concern. I would hope that a brief comment on the issue be included in the manuscript and the figures be included in supplemental material.

The supplementary analyzes and figures we have added (see our response to comment #1) should satisfy the referee as they are more detailed than the ones we sent in our previous response. These elements are referred to in the main text, which mentions the possible limitations of our method (lines 271-274).

2. The authors have missed the point of my concern regarding inactive/degraded elements.

I think the impact should be considered, but as the authors state in their letter, "This indicates that we did not consider active versus inactive elements in the analysis." I feel that making the claims that they do about the partial reconstruction would require having a model of the evolutionary topology of the elements, which the authors do not have. Preferring not to comment on this issue in the paper is odd.

This is because we did not perceive that the issue related to the "partial reconstruction" (of TE protein sequences). We actually thought the reviewer was concerned that Ka/Ks was overestimated due to TE degradation, undermining some of our conclusions about the absence of purifying selection.
We assume that the reviewer’s concern relates to this sentence "*This procedure effectively reconstructs (partial) homologous protein-coding sequences from a pair of TEs and investigates traces of selection that acted on these sequences before their deactivation*". We are not entirely sure what the reviewer means by "the claim that they do", but we believe that the concern results from a misunderstanding of the sentence we quoted above.
As opposed to what this sentence suggests, we do not infer the action of selective forces during a specific part of the TEs evolution. We just meant that selection only acts on functional TEs. We now realize that this statement is a given and that it is unrelated to our method. We have thus removed this sentence.
In effect, our method cannot distinguish between mutations that occurred before or after TE protein inactivation, as it would be extremely difficult to do so, especially at this scale (this would indeed require an evolutionary topology of TEs). We now make clear that pairwise Ka/Ks ratios represent the whole divergence of TE copies (lines 273-275): "*they do not account for changes in selection regimes during TE evolution, in particular the relaxing of selection after the potential TE deactivation*". We also make clear that traces of selection can be masked by subsequent mutations accumulating after TE deactivation: "*at least one of the homologous TE copies of a pair may be deactivated by a mutation during part of their divergence. Subsequent mutations on this copy should all be neutral, hence move the Ka/Ks ratio toward unity, even though the initial divergence between the TEs occurred under some form of selection*" (supplementary text lines 96-98).

Although we cannot distinguish active TEs, the possible impact of TE inactivation on Ka/Ks is addressed in our new analysis detailed in the supplementary section "*Impact of the pairwise approach on the reliability of the Ka/Ks analysis*", where we use overall TE sequence divergence as proxy for the time since TE deactivation.

3. If the authors do not wish to revise the text for the description of "Hit group evaluation", I would strongly recommend providing in the supplemental material a pseudocode representation of the process, to go with a diagramatic representation of the HTT system being assessed in supp figure 4.

We have added the pseudocode representation in a new supplementary section "*Pseudocode representation of the procedure that counts independent HTT events*". The pseudocode remains relatively close to natural language as we aimed at helping readers to understand our procedure rather than helping programmers to implement this procedure in code. We still think that the pseudocode section can achieve both goals, and we have added many comments on our R script.
We tried to produce a diagrammatic representation of the HTT events shown on the supplementary figure, but we failed to come up with a diagram that was useful. While the pseudocode is relatively simple, it led to a complicated diagram that we thought was more confusing than helpful. Below, we show our unfinished attempt, which is already quite complicated even though it does not represent all the steps.

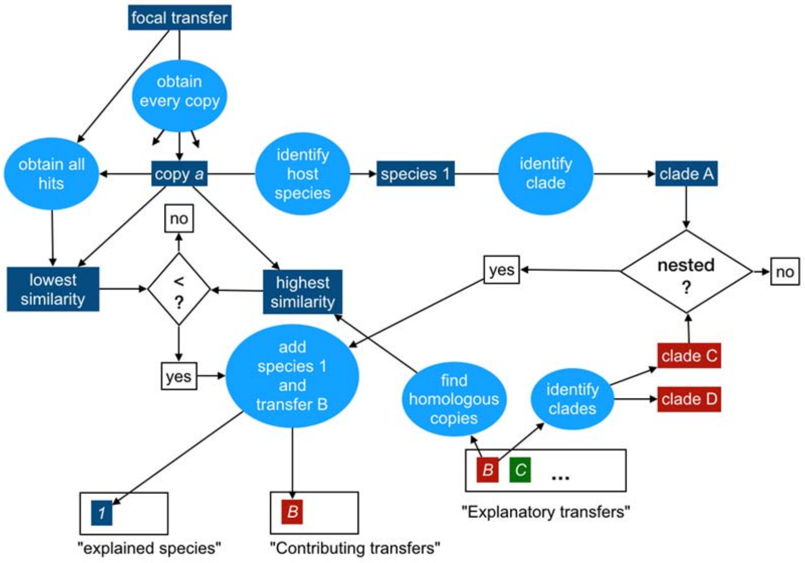

This diagram may be improvable, but we do not see how it could be made simple enough to be understood more easily than the pseudocode, and not be redundant with supplementary Figure 4 (now figure 8) at the same time. We believe that, given the relative simplicity of the pseudocode, the supplementary figure is sufficient. We think that, thanks to the reviewer input, the procedure can be understood and reproduced with the use of the dedicated section of the main text, the pseudocode representation, the supplementary figure and the R script. Adding a diagrammatic figure seems excessive, given that his procedure is not particularly central to the study.

4. Accepted.

5. I remain unable to have time to review the code because of the lack of clear structure and formatting. While the manuscript is not a methodological paper, in computational analysis, the devil is in the details. It is very troubling that the analysis is not presented in a form that would be understood to allow perfect replication of the analysis and for others to clearly read the approach.

We agree with the reviewer's assessment of our code. We initially thought that our code just needed to be made freely available. Had we realized that our code would be reviewed, we would have provided a better-formatted code.
We have reformatted all the scripts to more closely follow recommended R coding practices. We have restructured scripts into clearly distinct sections and added explanatory comments.
We have also changed some variable names to more explicit names, but we could not change all names to follow the best practices, as it might have added bugs. We cannot debug the whole pipeline due to the time it would take to run it (many weeks).

We now believe that the pipeline is presented in a form that, in complement to the manuscript, allows the analysis to be understood for replication. We however stress that, due to its mere length, it is not possible to review all the code in due time, regardless of its clarity. Granted, most of the code is not a result of this study, as it amounts to automation of certain tasks that can be reproduced by reading the method section.
One piece of code however is more original and useful in other contexts: it is the script that permits the computation of Ka and Ks on pairs of TEs. For this script, we have provided a demo dataset for the referee to run and review the code. The instructions to run this script are in the readme file of the GitHub repository.
It was not possible to provide demo datasets for other scripts, as these scripts can only produce meaningful results on large datasets of several GB.

We thank the reviewer for pointing these issues, thereby helping us to improve the paper and our coding practices.

Reviewer #2 (Remarks to the Author):

The authors have done an excellent job addressing my concerns. I appreciate their diligence. I have no additional comments and recommend acceptance of the manuscript.

Reviewer #3 (Remarks to the Author):

The authors have responded constructively to my comments, but I still have a concern regarding the Ka/Ks analysis within genomes. My original concern was that Ka/Ks measurements for TEs within a genome are not appropriate as per the Kryazhimskiy & Plotkin paper doi:10.1371/journal.pgen.1000304) . The authors have responded that this is not an issue because the comparisons made are not between alleles, but this misses the point. The Kryazhimskiy paper specifically talks about comparisons within bacterial species populations as being inappropriate and bacteria are prokaryotic, hence a between allele comparison is impossible.

We are sorry if our argument was not clear. We used the term "allele" to explain that we did not investigate polymorphism, which appeared to be Dr Adelson's concern. We did not mean to only refer to diploid organisms (the word allele is sometimes used to denote intraspecific variation in prokaryotes).
As TE copies at different loci are strictly paralogs, the vast majority of mutations that distinguish them are fixed in the host species, just like mutations between orthologs of different species. However, whether TE mutations are fixed in host species be independent of the Ka/Ks ratios we measure, as these reflect selection at the TE level, not at the host level (see our next reply).

My concern is that individual TEs in a genome are like individual bacterial clones in a population in that they arise at various times in a stochastic fashion.

I have taken the liberty of reproducing a relevant paragraph from the Kryazhimskiy paper below:

"The dN/dS ratio was originally developed for the analysis of genetic sequences from divergent species [1],[4],[5], the differences amoung which represent fixation events along independent lineages. Theoretical work on the relationship between dN/dS and selection likewise assumes that sequences are sampled from independent, divergent species [3], as do computer packages used to estimate dN/dS from data [6],[7]. Nonetheless, the dN/dS ratio test is frequently applied to data that may represent samples from a single population, particularly in the case of microbes (e.g. [3], [8]–[18]). In such cases, the differences between sequences do not represent fixation events along independent lineages, but rather polymorphisms segregating in a single population."

Dr Adelson is right in that we investigate selection among a "population" of TE copies (at different loci of a genome), not selection among host individuals that carry different variants, as opposed to what is done for all other types of genes. We now make clear (lines 258-262) what type of selection is at play. As we study selection among TE lineages and not host lineages, homologous TEs at different loci can indeed be seen as lineages of a clonal species parasitizing genomes, like bacterial clones may parasitize hosts.

There are additional points made in the paper that raise concerns about using dN/dS within populations, but I am not going to copy and paste the whole thing into this review. The situation described in this paragraph would seem to be equivalent to the situation of individual TEs within a genome. In this case, the Ka/Ks ratio is going to be insensitive to selection and should not be relied upon. I do not think it is appropriate to rely on this method of measuring selection for TEs within a genome/species. Therefore, I recommend that the results based on this approach be removed from the paper.

As Dr Adelson rightfully points, our interpretation of Ka/Ks ratios must account for the limitations of a within-population Ka/Ks analysis. We however respectfully disagree with his statement that Ka/Ks can be "insensitive to selection".

Kryazhimskiy and Plotkin (2008) warn against inferring relaxed selection when Ka/Ks ratios are high within a population, not against inferring negative selection when Ka/Ks ratios are low. Their study shows that Ka/Ks values are higher within a population than between species for the same intensity of negative selection. In that sense, Ka/Ks in a population is only *less* sensitive to selection (the authors use "*relatively* insensitive to selection"). What matters is that the same value of Ka/Ks (when it is below 1) corresponds to a stronger regime of negative selection when it is measured within a population than between species (see Figure 1 of Kryazhimskiy and Plotkin). Hence, it is entirely appropriate to infer negative selection within the same species when Ka/Ks < 1. Several studies that quote Kryazhimskiy and Plotkin have done such inference (e.g., (He, Sebaihia, Lawley et al. 2010, Castillo-Ramírez, Harris, Holden et al. 2011, Holt, Thieu Nga, Thanh et al. 2013)).
Furthermore, the reduced sensitivity of Ka/Ks to selection within a population reflects the short timescale of divergence between sequences. In our case, the overall molecular divergence between TE copies (~10% on average) is much larger than what is typically measured between variants of a population (< 1%), leaving more time for selection to be detectable.
We have therefore maintained our interpretations about varying intensity of purifying (negative) selection among TEs from different types. Kryazhimskiy and Plotkin's results (figure 1) show that Ka/Ks ratios decrease with the strength of negative selection in the range of Ka/Ks ratios we observe (between 0.3 and 1). We think it is valid to suggest that purifying selection is weaker in Jockey TEs and stronger in LTR elements, which are the two extremes we discuss among retrotransposons.
We however account for Kryazhimskiy and Plotkin's caution about inferences based on high Ka/Ks ratios. To this effect, we have added a supplementary section "*Ka/Ks and selection acting on transposable elements*" to explain that our power to detect selection is limited. In the main text, we say that this limitation may apply to Jockey elements (lines 295-296). We still argue (lines 296-299) that the mechanism by which DNA transposons are recognized by transposases leaves no possibility for a form of selection based on the synonymy of mutations.

This comment led us to rework most of the section "*Selective constraints acting on transposable elements*". We realized that it made more sense to first discuss TEs that diverged within genomes before addressing divergence of TE via horizontal transfer. As a result, we have modified Figure 4 so that upper density curves represent Ka/Ks ratios between copies of the same genome. The values remain unchanged, of course.

I have one additional comment with respect to the author's response to my query about BovB HTT between mammals and other taxa. Our original method (Ivancevic et al) for detecting BovB HTT was quite stringent and used patchy phylogenetic distribution as one of the criteria because this is a hallmark of HTT. Ascertainment of a patchy distribution requires that no trace of the TE in question can be detected in this genome. While it is true that one cannot estimate the probability of complete removal of all traces of a TE, observations of persistent TE fragments from "fossil" TEs are extremely commonplace so it is reasonable to conclude that complete removal of a particular TE family/subfamily from a genome has an extremely small probability. If the large discrepancy between our results for BovB and the authors' results for BovB primarily differ due to the use of a patchy distribution, then this is something that should be discussed as it represents a profound difference in methodology, particularly since the highly patchy distribution of BovB makes them a useful validation set for other methods. We reported in excess of 50 high confidence HTT events for BovB involving mammalia in our supplementary material so the real question is why are these undetected? Exploration of these discrepancies should result in the authors being able to make their method more sensitive. I stand by my earlier recommendation that this still needs to be discussed.

We did not mean to imply that patchiness of TEs was an unreliable criterion. We just meant that we cannot automatically apply this criterion to our dataset. It is difficult to assess the particular influence of this criterion on the differences between our studies, as the approaches differ in so many respects. We now discuss these differences in a new supplementary section "*Lower number of HTT involving mammals compared to previously published results*". Briefly, we raise the fact that: we only detected TE homology at the DNA level, we applied filters based on the Ks distribution of TEs, our clustering procedure was possibly more conservative, so was our criterion to reduce the risk of DNA contamination. Here we show that relaxing some of the filters we used increases the number of BovB hit groups between mammals and squamates.
We still favor conservativeness over sensitivity because we cannot check the reliability of each inferred transfer events. Changing our method may give a more accurate number of HTT between

mammals and other vertebrates, but it is difficult to anticipate the effects of such change on the reliability of HTT between other lineages, namely ray-finned fishes. We also cannot loosen the filters that we applied before the clustering of hits, as the resulting number of hits would be too large for our clustering procedure.

This reply submitted by David Adelson of the University of Adelaide

We thank Dr Adelson for his comments that helped us to improve the interpretation of our results.

Castillo-Ramírez, S., S. R. Harris, M. T. G. Holden, et al. (2011). "The Impact of Recombination on dN/dS within Recently Emerged Bacterial Clones." PLOS Pathogens **7**(7): e1002129. www.doi.org/10.1371/journal.ppat.1002129

He, M., M. Sebaihia, T. D. Lawley, et al. (2010). "Evolutionary dynamics of Clostridium difficile over short and long time scales." Proceedings of the National Academy of Sciences **107**(16): 7527. www.doi.org/10.1073/pnas.0914322107

Holt, K. E., T. V. Thieu Nga, D. P. Thanh, et al. (2013). "Tracking the establishment of local endemic populations of an emergent enteric pathogen." Proceedings of the National Academy of Sciences **110**(43): 17522. www.doi.org/10.1073/pnas.1308632110

Kaehler, B. D., V. B. Yap and G. A. Huttley (2017). "Standard Codon Substitution Models Overestimate Purifying Selection for Nonstationary Data." Genome biology and evolution **9**(1): 134-149. www.doi.org/10.1093/gbe/evw308

Kryazhimskiy, S. and J. B. Plotkin (2008). "The Population Genetics of dN/dS." PLOS Genetics **4**(12): e1000304. www.doi.org/10.1371/journal.pgen.1000304

REVIEWERS' COMMENTS:

Reviewer #1 (Remarks to the Author):

I would like to thank the authors for so rigorously addressing the issues that I have raised during review.

The manuscript describes interesting and valuable findings and I feel that it should be considered for publication.

Reviewer #3 (Remarks to the Author):

I would like to thank the authors for taking all of my suggestions seriously and working hard to revise this manuscript. I can see that they have put in a lot of work to revise the paper and I think it is a much better paper as a result of their efforts.

I feel that they have addressed all of my comments appropriately and I am prepared to recommend that this paper be accepted in its present form.

David Adelson
University of Adelaide
